# Investigating and correlating photoelectrochemical, photocatalytic, and antimicrobial properties of $\hbox {TiO}_2$ nanolayers

**DOI:** 10.1038/s41598-021-01165-x

**Published:** 2021-11-12

**Authors:** Volker Seiß, Uta Helbig, Ralf Lösel, Maik Eichelbaum

**Affiliations:** 1 Faculty of Applied Chemistry, Georg Simon Ohm University of Applied Sciences Nuremberg, 90489 Nuremberg, Germany; 2 Faculty of Materials Engineering, Georg Simon Ohm University of Applied Sciences Nuremberg, 90489 Nuremberg, Germany

**Keywords:** Electrochemistry, Materials chemistry, Photochemistry, Physical chemistry

## Abstract

Semiconducting transition metal oxides such as $\hbox {TiO}_2$ are promising photo(electro)catalysts for solar water splitting and photoreduction of $\hbox {CO}_2$ as well as for antibacterial, self-, water and air-cleaning coatings and admixtures in paints, building materials, on window glass or medical devices. In photoelectrocatalytic applications $\hbox {TiO}_2$ is usually used as photoanode only catalyzing the oxidation reaction. In coatings and admixtures $\hbox {TiO}_2$ works as heterogeneous catalyst and has to catalyze a complete redox cycle. While photoelectrochemical charge transport parameters are usually quite well accessible by electrochemical measurements, the quantitative description of photocatalytic properties is more challenging. Here, we present a systematic structural, photoelectrocatalytic, photocatalytic and antimicrobial study to understand if and how photoelectrochemical parameters can be used to predict the photocatalytic activity of $\hbox {TiO}_2$. For this purpose $\hbox {TiO}_2$ thin films on flourine-doped tin oxide substrates were prepared and annealed at temperatures between 200 and 600 $^{\circ }\hbox {C}$. The film morphologies and thicknesses were studied by GIXRD, FESEM, and EDX. Photoelectrochemical properties were measured by linear sweep voltammetry, photoelectrochemical impedance spectroscopy, chopped light chronoamperometry, and intensity modulated photocurrent/ photovoltage spectroscopy. For comparison, photocatalytic rate constants were determined by methylene blue degradation and Escherichea coli inactivation and correlated with the deduced photoelectrocatalytic parameters. We found that the respective photoactivities of amorphous and crystalline $\hbox {TiO}_2$ nanolayers can be best correlated, if the extracted photoelectrochemical parameters such as charge transfer and recombination rates, charge transfer efficiencies and resistances are measured close to the open circuit potential (OCP). Hence, the interfacial charge transport parameters at the OCP can be indeed used as descriptors for predicting and understanding the photocatalytic activity of $\hbox {TiO}_2$ coatings.

## Introduction

At least since the pioneering work of Fujishima and Honda in 1972^1,2^ semiconducting transition metal oxides such as titanium dioxide ($\hbox {TiO}_2$) are intensively studied as photoanode and photocatalyst materials for photosplitting of water with ultraviolet (UV) light^3^. Recently reported record-high quantum efficiencies between 81.8% at 437 nm and 3.2% at 1000 nm of N-$\hbox {TiO}_2$ even opens up completely new vistas to utilize nearly the whole solar spectrum beyond the UV range for photo(electro)catalytic applications^4^. Moreover, $\hbox {TiO}_2$ can photocatalytically reduce $\hbox {CO}_2$ to useful chemicals under sun light irradiation^5,6^ making it a promising platform for a carbon-neutral energy and raw material economy. Photocatalytic water splitting by $\hbox {TiO}_2$ induces highly reactive intermediate species such as hydroxyl radicals (HO$^*$), hydroperoxyl radicals (HOO$^*$), and superoxide radical anions (O$_2^{*-}$)^7^, whose photo(electro)catalytic generation can be described by the following equations:

1 $$\begin{aligned}&\hbox {TiO}_{2} + \hbox {h}\nu \longrightarrow \hbox {h}_{\mathrm{VB}}^{+} + \hbox {e}_{\mathrm{CB}}^{-} \end{aligned}$$

2 $$\begin{aligned}&\hbox {H}_{2}\hbox {O} + \hbox {h}_{\mathrm{VB}}^{+} \longrightarrow \hbox {HO}^{*} + \hbox {H}^{+} \end{aligned}$$

3 $$\begin{aligned}&\hbox {O}_{2} + \hbox {e}_{\mathrm{CB}}^{-} \longrightarrow \hbox {O}_{2}^{*-} \end{aligned}$$

4 $$\begin{aligned}&\hbox {O}_{2}^{*-} + \hbox {H}^{+} \longrightarrow \hbox {HOO}^{*} \end{aligned}$$

Here $\hbox {h}\nu$ denotes the irradiation energy, $\hbox {h}_{\mathrm{VB}}^{+}$ a photogenerated hole in the valence band of $\hbox {TiO}_2$, and $\hbox {e}_{\mathrm{CB}}^{-}$ a photoexcited free electron in the conduction band. The thus formed radical species are characterized by very high standard reduction potentials, which can hence effectively oxidize organic or biologic components to $\hbox {CO}_2$ and $\hbox {H}_{2}\hbox {O}$. As a consequence, this property of $\hbox {TiO}_2$ enables many more interesting applications. One example is the photocatalytic treatment of waste water with disperged or immobilized $\hbox {TiO}_2$ under UV light^8,9^. Moreover, glassy surfaces coated with a polycrystalline titania film exhibit excellent anti-fogging and self cleaning properties under irradiation^10^. In addition to these super-hydrophilic properties, the photocatalytic oxidation of organic and inorganic contaminations and the antimicrobial effect of titanium dioxide have been utilized to develop materials and surfaces avoiding the coverage by algae, moss, mold, bacteria etc.^10–13^ Additionally, photoactive building materials such as cobblestones, roof tiles and concrete walls containing or coated with $\hbox {TiO}_2$ are being tested in street tunnels to photocatalytically reduce the $\hbox {NO}_{\mathrm{x}}$ content of ambient air^14^. $\hbox {TiO}_2$ is one of the most promising candidates as catalyst for the aforementioned applications, because it is characterized by a high exciton binding energy, it is unsoluble in aqueous liquids, chemically and biologically inert, photostable, not toxic, relatively cheap and abundant^8,15,16^. The various modifications of $\hbox {TiO}_2$ can be prepared in many different forms such as nanoparticles, nanotubes, nanofibers, nanocubes etc., but also as composite and hybride material - e.g. in combination with polymers or carbon nanomaterials - by comparably cheap methods^17^. Layers of $\hbox {TiO}_2$ have been prepared, e.g., by sol–gel spin- or dip coating methods, thermal oxidation of titanium sheets, spray pyrolysis, chemical vapor deposition, pulsed laser deposition, or magnetron sputtering techniques^8,18^.

There is broad agreement in the scientific community that the photocatalytic reaction rate for the different reaction schemes is adversely affected by a radiative or non-radiative recombination of photoexcited charge carriers^18–22^. However, it is very often not clear, if a fast electron-hole recombination or a slow charge diffusion to the reactive surface is the limiting factor for the reaction rate. Most known forms of $\hbox {TiO}_2$ are characterized as n-type semiconductors, which is often attributed to an easy release of oxygen leaving electrons as majority charge carriers in the lattice. These electrons can be trapped in the lattice as $\hbox {Ti}^{3+}$ and released again as $\hbox {Ti}^{4+}$(lattice) + $\hbox {e}_{\mathrm{CB}}^{-}$. Such donor sites can also act as effective electron-hole recombination center. On the other hand, photoexcited holes can be trapped as $\hbox {O}^{-}$ in the lattice and released again as $\hbox {O}^{2-}$(lattice) + $\hbox {h}_{\mathrm{VB}}^{+}$. The $\hbox {O}^{-}$ site is also considered as possible recombination center. However, shallow donor sites for electrons (or acceptor sites for holes) with energies just below the conduction band minimum (just above the valence band maximum), where thermal energy is sufficient to lift the charge carriers into the bands, may give rise to a fast charge transfer process. In contrast, deep donor or acceptor states decrease the charge transfer rate since the electron hopping between deep traps is usually slower than migration through the conduction band and shallow traps, thus enhancing the possibility for a charge carrier recombination process.

**Figure 1:**
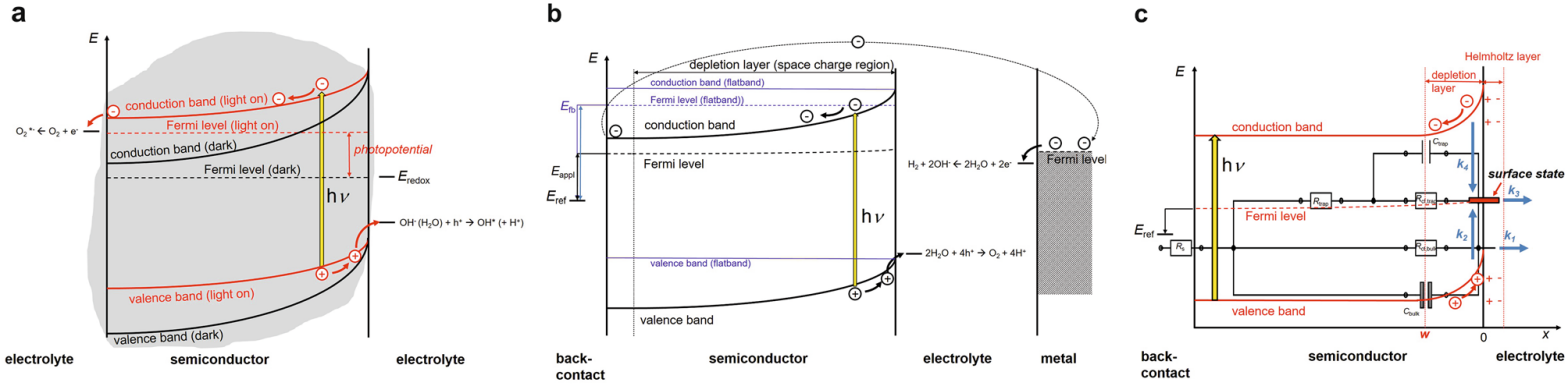
Energy schemes for a photocatalytically active n-type semiconductor particle (**a**) and a photoelectrochemical cell with an n-type semiconductor electrode as photoanode (**b**) with depletion layer, interfacial semiconductor-electrolyte charge transfer, and possible redox reactions. (**c**) Schematic band diagram with charge transfer rate constants and equivalent circuit elements used to fit EIS data.

In Fig. 1 energy diagrams of a photoexcited n-type semiconductor with depletion zone for photocatalytic and photoelectrocatalytic processes, respectively, as described by Gerischer^23^, and the most important transfer and recombination processes with their corresponding rate constants as suggested by Ponomarev and Peter^24^ are schematically summarized. In this model the absorption of light induces a flux of holes toward the semiconductor-electrolyte interface and of electrons to the back-contact (or to the other side of the semiconductor particle). The charge transfer efficiency is limited by a competition between hole transfer from the semiconductor to the electrolyte and the recombination of holes with electrons from the conduction band. Holes can be directly transferred via the valence band (with rate constant $k_1$), react with electrons in surface states ($k_2$), or can be transferred to the electrolyte via surface states ($k_3$). Electrons can be transferred via the conduction band to the back-contact or they can be captured in surface states with rate constant $k_4$ acting again as recombination sites. In addition, direct band to band electron-hole recombination or recombination at defects in the bulk are possible, but usually less likely than surface recombination. The complexity increases even more, if different phases, dopants, artificial defects etc. are to be included^3^.

Many attempts have been made to study and quantify the aforementioned charge transport processes. With pump-probe spectroscopy the charge generation and recombination processes in $\hbox {TiO}_2$ where analyzed on a femtosecond time scale^20,25^. However, it turned out that the high photon flux of a femtosecond pulse induced second order kinetics and very fast recombination rates that might not reflect the situation in a semiconductor under “normal” light intensities. Alternatively, the measurement of transient photocurrents with chopped light chronoamperometry (CLCA) under potentiostatic conditions was utilized to deduce surface recombination rate constants. Moreover, photoelectrochemical methods such as intensity modulated photocurrent spectroscopy (IMPS), intensity modulated photovoltage spectroscopy (IMVS), and photoelectrochemical impedance spectroscopy (PEIS) under controlled light intensity radiation proved to be powerful tools for deducing electron and hole lifetimes and rate constants for charge transfer and recombination^21,22,23–26^. Apart from these electrochemical methods the photoactivity of semiconducting powders and films has been very often analyzed by photometrically monitoring the photocatalyzed degradation of organic dyes such as methylene blue^27^. An established procedure to describe the antimicrobial photoactivity comprehends the determination of the inactivation rate for bacterial cultures on the surface of a photocatalyst under irradiation^28,29^.

However, direct links rationally connecting photoelectrochemical, photocatalytic, and antimicrobial properties of $\hbox {TiO}_2$ coatings are still missing and shall be provided by the present work. We hence prepared $\hbox {TiO}_2$ films via a well reproducible hydrothermal sol–gel route and a spin-coating process. Quartz glass slides coated with fluorine-doped tin oxide (FTO) were used as substrates to realize a sufficient conductivity for photoelectrochemical measurements. Samples were annealed at temperatures between 200 and 600 $^{\circ }\hbox {C}$ to obtain various amorphous and crystalline titania phases with different photo(electro)catalytic properties.The morphology and crystallinity of the films was analyzed by grazing incidence X-ray diffraction (GIXRD) and field emission scanning electron microscopy (FESEM) in combination with energy dispersive X-ray spectroscopy (EDX). The photoactivity of the samples towards organic and biologic counterparts under UV light was measured by the degradation of methylene blue (MB) and inactivation of Escherichia coli (E. coli) bacteria, respectively. Degradation and inactivation rates were correlated with kinetic and electrochemical parameters as obtained by IMVS, IMPS, PEIS, linear sweep voltammetry (LSV), and CLCA.

## Results

### Structural characterization

#### XRD analysis

**Figure 2:**
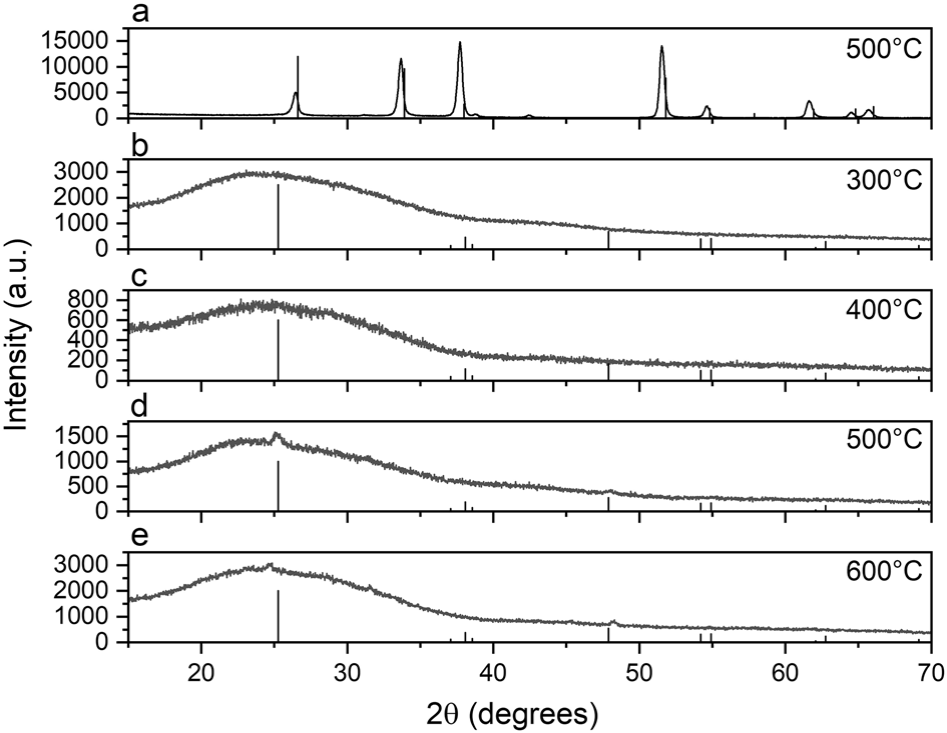
GIXRD patterns of $\hbox {TiO}_2$ thin films coated on the FTO side (**a**) and on the reversed non-FTO coated side of a quartz glass (**b**–**e**), respectively. Samples were calcined at the indicated temperatures. The diffraction pattern of the $\hbox {SnO}_2$ modification cassiterite (**a**) and the $\hbox {TiO}_2$ modification anatase (**b**–**e**), respectively, are displayed as vertical lines for comparison.

The structural characterization of nanolayers on crystalline substrates is usually a challenging task due to the very low signal intensities and strong interferences with the dominating substrate. We hence chose to execute GIXRD to increase the surface sensitivity of the measurement. However, it turned out that the reflections due to the cassiterite structure of the FTO sublayer still dominated the whole diffractogram making it impossible to deduce any information on the $\hbox {TiO}_2$ layers (Fig. 2a).

We thus alternatively prepared $\hbox {TiO}_2$ nanolayers annealed at 300, 400, 500, and 600 $^{\circ }\hbox {C}$, respectively, after the same protocol as described for the FTO-coated samples, but with the exception that the layers were spin-coated on the opposite, i.e. FTO-free side of the glass slides. Now, a very broad signal peaking at about $24^{\circ }$ with a broad shoulder at about $42^{\circ }$ can be observed in the diffractograms—features that are characteristic for the amorphous silica phase of the quartz glass substrate (Fig. 2b–e). As for the $\hbox {TiO}_2$ nanolayers annealed at 300 $^{\circ }\hbox {C}$ and 400 $^{\circ }\hbox {C}$, respectively, no further unambigous features or peaks could be identified. In contrast, two small but distinct reflections peaking at $25.1^{\circ }$ and $48.0^{\circ }$, respectively, appear in the diffractogram of the sample annealed at 500 $^{\circ }\hbox {C}$. These peaks were compared with the reflections expected for the three polymorphs of $\hbox {TiO}_2$: anatase, rutile, and brookite. The tetragonal anatase phase exhibits its three most intense signals at $2\theta$ angles of $25.2^{\circ }$ (major), $37.7^{\circ }$, and $47.9^{\circ }$ (JCPDF No. 21-1272). The tetragonal rutile phase is characterized by the three most intense reflections at $27.4^{\circ }$ (major), $35.9^{\circ }$, and $54.3^{\circ }$ (JCPDF No. 34-0180), and the orthorhombic brookite phase typically shows three reflections with nearly similar intensities at $25.4^{\circ }$, $25.7^{\circ }$, and $30.8^{\circ }$ (JCPDF No. 29-1360). Thus, the GIXRD pattern of the $\hbox {TiO}_2$ nanolayer can be best explained by its assignment to the anatase phase, though only the two most intense peaks corresponding to the (101) and (211) crystal planes can be resolved. In the 600 $^{\circ }\hbox {C}$ sample two peaks at 24.8 $^{\circ }\hbox {C}$ and 48.3 $^{\circ }$ are observed, slightly shifted with respect to the reflections of the 500 $^{\circ }\hbox {C}$ sample. Again, this diffractogram can be explained best by assigning it to the anatase structure. However, the shift of the (101) reflex from the expected 25.2 $^{\circ }$ to 24.8 $^{\circ }$ suggests a strain of the elementary cell in the c-direction. Klaysri et al. reported on a small shift of XRD signals in nanocrystalline anatase upon high temperature treatment in $\hbox {N}_2$ atmospheres, which was explained by the incorporation of N atoms into the $\hbox {TiO}_2$ crystal structure accompanied by a distortion of the $\hbox {TiO}_6$ octahedron^30^. However, the authors reported on a shift to higher $2\theta$ angles, whereas we observed a shift to lower angles. Rajender and Giri described a broadening and a slight shift of the (101) reflex of anatase to lower angles in the X-ray diffractogram of ball-milled $\hbox {TiO}_2$ nanocrystals^31^ and assigned this to milling-induced tensile strain in the nanocrystals. This shift was accompanied by the appearance of two additional peaks at 31.3 $^{\circ }$ and 41.6 $^{\circ }$, which were attributed to the (112) and (312) planes of a newly formed oxygen-deficient $\hbox {Ti}_3\hbox {O}_5$ phase (JCPDS No. 74-0819). A closer look at the diffractogram of the $\hbox {TiO}_2$ nanolayer annealed at 600 $^{\circ }\hbox {C}$ of our work reveals a weak reflex at 31.5 $^{\circ }$. It remains speculative if a strained anatase accompanied by the formation of a $\hbox {Ti}_3\hbox {O}_5$ phase can also be the explanation for the observed XRD pattern of the 600 $^{\circ }\hbox {C}\ \hbox {TiO}_2$ nanolayer investigated in our work, since the expected second peak in the range of 41 $^{\circ }$–42 $^{\circ }$ could not be identified. However, it seems reasonable that a high annealing temperature cannot only induce a phase change from amorphous $\hbox {TiO}_2$ to crystalline anatase, but that it can also introduce tensile stress and the formation of oxygen-deficient phases in $\hbox {TiO}_2$ nanolayers coated on quartz glass substrates.

#### SEM analysis

**Figure 3:**
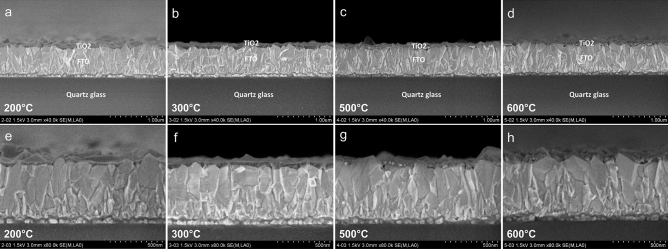
Cross-sectional FESEM images of $\hbox {TiO}_2$ -coated FTO glass slides calcined at temperatures indicated in the images recorded with 40.0 k magnification (**a**–**d**) and corresponding images with 80.0 k magnification (**e**–**h**).

In order to elucidate the morphology and film thickness of the $\hbox {TiO}_2$ nanolayers, cross-sections of the samples annealed at 200, 300, 500, and 600 $^{\circ }\hbox {C}$ were prepared and investigated by FESEM. In addition, EDX elemental maps were recorded to identify the elemental distribution in the cross-sections. Figure S1 shows the scanning electron micrograph of the $\hbox {TiO}_2$ coated FTO glass calcined at 200 $^{\circ }\hbox {C}$ and the corresponding EDX elemental maps for Si, Sn, and Ti with a magnification of 50.000. The Si map clearly indicates the quartz glass and the Sn map the dimension of the FTO layer. Only a very weak contrast in the Ti map above the FTO layer hints at the $\hbox {TiO}_2$ nanolayer. In the SEM images recorded with the higher magnification of 80.000 the glass substrate, the FTO sublayer, and the $\hbox {TiO}_2$ coating can be unambigously distinguished (Fig. 3).

It becomes also obvious that the morphology of the FTO layer is rather rough. However, the substrate is completely covered by a continuous $\hbox {TiO}_2$ film. The $\hbox {TiO}_2$ layer exhibits a thickness of about 100-150 nm for the sample annealed at 200 $^{\circ }\hbox {C}$, and of about 50–100 nm for the 300 $^{\circ }\hbox {C}$ film. Upon annealing at 500 $^{\circ }\hbox {C}$ the $\hbox {TiO}_2$ layer adjusts to the roughness of the underlying FTO substrate and the thickness decreases to about 50 nm. As for the 600 $^{\circ }\hbox {C}$ sample the $\hbox {TiO}_2$ film can be hardly distinguished from the FTO sublayer shrinking to thicknesses of approximately 50 nm or less. This shrinking of the film and the increased roughening can be explained by the progressing crystallination process with higher temperatures and would also be in agreement with the temperature-induced strain on the 600 $^{\circ }\hbox {C}\ \hbox {TiO}_2$ nanocrystal lattice as postulized for the interpretation of the GIXRD results.

### Photocatalytic activity

**Figure 4:**
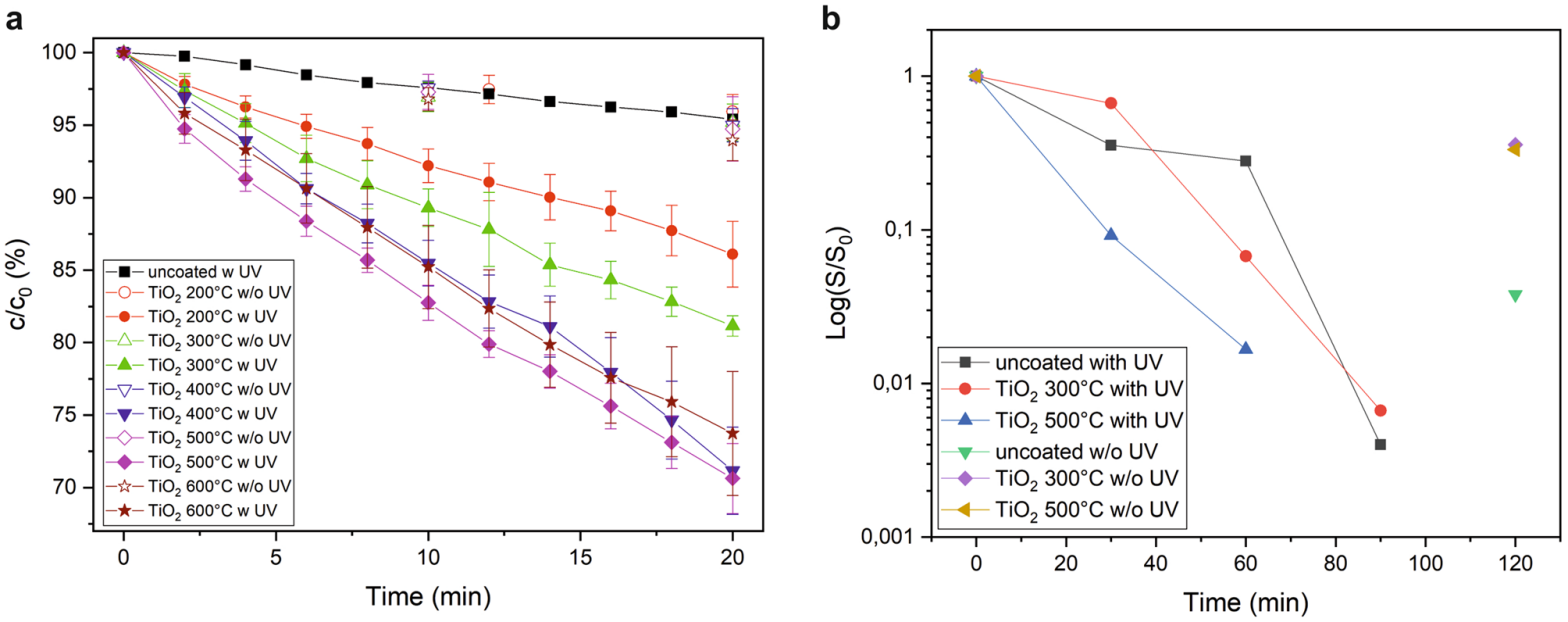
(**a**) Methylene blue degradation kinetics expressed as ratio between actual (*c*) and starting concentration ($c_0$) versus time on neat and $\hbox {TiO}_2$-coated FTO glass slides with and without 369 nm irradiation. Data points indicate the mean of three measurements and error bars denote the standard error of the mean. (**b**) Bacterial inactivation kinetics expressed as logarithm of survival rates versus time on neat and $\hbox {TiO}_2$-coated FTO glass slides with and without UV-A irradiation. The data point of the $\hbox {TiO}_2$ 500 $^{\circ }\hbox {C}$ with UV sample at 90 minutes cannnot be shown in the logarithmic presentation since the survival rate approached zero. The drawn lines serve as guide to the eyes.

The investigation of the detailed molecular mechanism of the photocatalytic MB degradation was not the focus of our study. However, the $\hbox {TiO}_2/\hbox {UV}$ catalyzed degradation pathway of aqueous MB solutions was analyzed in detail by Houas et al. by investigating intermediates and final products with HPLC, LC-MS, and GC-MS^32^. Based on these findings they proposed that the chromophoric structure of MB is attacked by hydroxyl radicals formed by photoholes or photoelectrons of irradiated $\hbox {TiO}_2$ or by a direct attack of the organic reactant by photoholes leading to a complete mineralization of the dye molecule to the final products $\hbox {CO}_2$, $\hbox {H}_{2}\hbox {O}$, $\hbox {Cl}^{-}$, $\hbox {SO}_{4}^{2-}$, and $\hbox {NO}_{3}^{-}$.

The photocatalytic activity of our $\hbox {TiO}_2$ nanolayers was investigated by measuring the degradation rate of MB dye under 369 nm light irradiation at $1000\,\hbox {W}/\hbox {m}^2$ and in the dark. The MB degradation curves for all measured samples are summarized in Fig. 4a. The uncoated FTO glass showed after 20 minutes a small but distinct reduction of the initial MB concentration by 5%. All $\hbox {TiO}_2$-coated samples exhibited a significantly larger MB degradation. The strongest effect was observed for the $\hbox {TiO}_2$ nanolayer annealed at 500 °C, closely followed by the 400 °C and 600 °C samples, with a reduction of the initial MB concentration after 20 minutes between 26 and 29%. The samples annealed at the lower temperatures of 300 and 200 °C, respectively, followed with a considerably lower MB degradation of about 19 and 14%, respectively. The MB degradation was also investigated in the dark. As a result, no sample achieved a reduction of the initial MB concentration by more than 6% on average after 20 minutes. Hence, the MB degradation can be clearly associated with a photocatalytic effect of $\hbox {TiO}_2$. The MB degradation rate $R_{\mathrm{MB}}$ was calculated for all samples by the following equation:

5 $$\begin{aligned} R_{\mathrm{MB}} = \frac{c_{0}-c_{\mathrm{final}}}{time}, \end{aligned}$$

with $c_0$ being the initial MB concentration and $c_{\mathrm{final}}$ the MB concentration after 20 minutes. The resulting degradation rates are summarized in Table S1 and reflect the same trend as already discussed before, i.e. with the highest rates observed for the 500, 400 $^{\circ }\hbox {C}$, and 600 °C samples, followed by the $\hbox {TiO}_2$ layers annealed at 300 and 200 $^{\circ }\hbox {C}$, respectively. The smallest value was calculated for the uncoated glass.

### Antimicrobial activity

The mechanism of bacterial inactivation by photocatalysts is mostly described by an inital attack of the cell membrane by photogenerated hydroxyl radicals leading to lipid peroxidation^29,33^. Subsequent oxidative attacks to intracellular components cause the final cell death. Wolfrum et al. could even prove a complete mineralization of E. coli bacteria to $\hbox {CO}_2$ and $\hbox {H}_{2}\hbox {O}$ by photocatalytic oxidation on $\hbox {TiO}_2$-coated surfaces^34^. Alternatively, inhibition of the cell respiration by photooxidation of coenzyme A has been suggested as reason for bacterial inactivation^35^.

The antimicrobial activity of uncoated and $\hbox {TiO}_2$-coated FTO glasses annealed at 300 and 500 $^{\circ }\hbox {C}$ under UV-A irradiation was tested by measuring the photocatalytic inactivation of E. coli bacteria. In prelimary tests a dilution step of 1:1000 for the E. coli overnight culture proved to be ideal to obtain a high but still countable number of cultures. This number was defined as $S_0$ value. All bacteria-treated glass slides exhibited a reduction of the number of bacteria cultures after irradation and plate pouring. Images of petri dishes with bacterial cultures treated under different experimental conditions are shown in Fig. S2 and the kinetic analyses of the inactivation is summarized in Fig. 4b.

As a result, a distinct photocatalytic antibacterial effect of the $\hbox {TiO}_2$ coatings could be observed. The $\hbox {TiO}_2$ film treated at 300 $^{\circ }\hbox {C}$ could reduce the survival rate to about $6.7\%$ after 60 minutes UV-A irradiation. The 500 $^{\circ }\hbox {C}$ sample exhibited even a survival rate of only $1.7\%$ after the same period of time. However, it becomes obvious that even the blind samples measured in the dark after 120 min indicated a significant bacterial inactivation to a maximum survival rate of about $36\%$. Obviously, the experiments with living material such as bacteria suffer from a challenging reproducibility making it difficult to deduce quantitative numbers and to compare samples with only slightly different properties. However, the superior photocatalytic effect of the $\hbox {TiO}_2$ thin film on FTO calcined at 500 $^{\circ }\hbox {C}$ on the degradation of E. coli relative to the uncoated and 300 $^{\circ }\hbox {C}\ \hbox {TiO}_2$ sample can be unambiguously identified supporting the trends already observed for the MB degradation kinetics.

### Electrochemical characterization

#### Linear sweep voltammetry (LSV)

**Figure 5:**
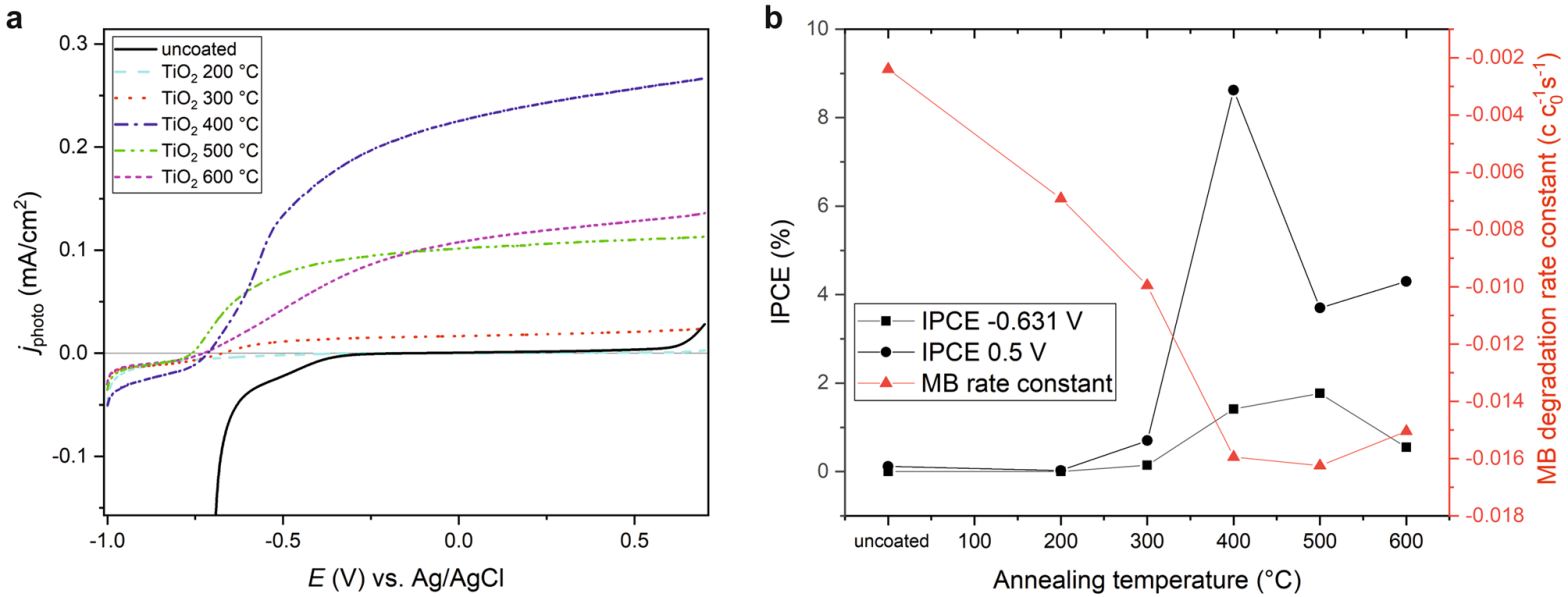
(**a**) Linear sweep voltammetric curves of neat and $\hbox {TiO}_2$-coated FTO glass slides recorded under 369 nm irradiation at $100\ \hbox {W}/\hbox {m}^{2}$. (**b**) Incident photon conversion efficiencies (IPCE) of the samples as calculated for the potentials of 0.5 V and $-0.631\,\hbox {V}$ versus Ag/AgCl, respectively, and methylene blue degradation rate constants for comparison. The drawn lines in (**b**) serve as guide to the eyes.

LSV measurements were performed for uncoated and $\hbox {TiO}_2$-coated FTO glasses to measure the photopotentiodynamic characteristics of the samples and to determine open-circuit potentials (OCP). Without irradiation no positive currents were recorded within the electrochemical potential range between $-1.0$ and 0.5 V (potenials were always measured versus Ag/AgCl reference) for all investigated samples. The results under 369 nm irradiation at a light intensity of $100\,\hbox {W}/\hbox {m}^{2}$ are summarized in Fig. 5a. The uncoated sample shows nearly no photocurrent in the investigated potential range. Only above the break-down potential of about 0.5 V a positive current is measured. The $\hbox {TiO}_2$-coated FTO glass annealed at 200 $^{\circ }\hbox {C}$ exhibits a very small photocurrent with an OCP of $-0.249\,\hbox {V}$. Significant photocurrents are measured for the 300, 400, 500 and 600 $^{\circ }\hbox {C}$ annealed $\hbox {TiO}_2$ samples. The OCP is shifted to more negative values with increasing annealing temperature, i.e. to $-0.672\,\hbox {V}$ (300 $^{\circ }\hbox {C}$), $-0,715\,\hbox {V}$ (400 $^{\circ }\hbox {C}$), and $-0.759\,\hbox {V}$ (500 $^{\circ }\hbox {C}$), respectively. Only annealing at 600 $^{\circ }\hbox {C}$ induces with an OCP of $-0.728\,\hbox {V}$ a small shift to a slightly more positive value with respect to the 500 $^{\circ }\hbox {C}$ sample. The direct comparison of the potential-dependent photocurrent characteristics exhibits remarkable differences. At high potentials above 0 V the photocurrents of the coated samples rank in the following order: 400 $^{\circ }\hbox {C}$ $\hbox {TiO}_2 > 600\,^{\circ }\hbox {C}$ $\hbox {TiO}_2 > 500\,^{\circ }\hbox {C}$ $\hbox {TiO}_2 >300\,^{\circ }\hbox {C}$ $\hbox {TiO}_2 > 200\,^{\circ }\hbox {C}$ $\hbox {TiO}_2$.

Moreover, the fill factor, defined as ratio between the area enclosed by the actual photocurrent and voltage in the voltammogram to the area of an ideal rectangle (where the photocurrent rises instantaneously to the maximum value at the OCP), is largest for the 500 $^{\circ }\hbox {C}$ sample followed by the 400 $^{\circ }\hbox {C}$ and 600 $^{\circ }\hbox {C}$ sample, respectively. The smaller the fill factor, the larger the influence of increased charge recombination as well as hindered charge transport and charge separation on the resulting photocurrent. This is also reflected by the order of the incident photon conversion efficiencies (IPCE) at 0.5 V as shown in Fig. 5b, which were calculated by the following equation:

6 $$\begin{aligned} \hbox {IPCE} {=} \frac{j_{\mathrm{photo}} \cdot \hbox {h} \cdot \hbox {c}}{I_{\mathrm{light}} \cdot \hbox {e} \cdot \lambda }, \end{aligned}$$

where $j_{\mathrm{photo}}$ is the photocurrent density, h the Planck constant, c the speed of light, $I_{\mathrm{light}}$ the light intensity, e the elementary charge, and $\lambda$ the irradiation wavelength. The 400 $^{\circ }\hbox {C}\ \hbox {TiO}_2$ sample shows at 0.5 V versus Ag/AgCl with 8.6% the highest IPCE, followed by the 600 $^{\circ }\hbox {C}$ and 500 $^{\circ }\hbox {C}$ sample with 4.3, and 3.7%, respectively. However, at potentials closer to the OCP this order changes. E.g., at $-0.631\,\hbox {V}$ the 500$^{\circ }\hbox {C}$ $\hbox {TiO}_2$ sample exhibits now the highest IPCE with 1.8%, followed by 400 $^{\circ }\hbox {C}\ \hbox {TiO}_2$ with 1.4%. At this potential the 600 $^{\circ }\hbox {C}\ \hbox {TiO}_2$ film shows only an IPCE of 0.5%.

The photocurrent $I_{\mathrm{photo}}$ is expected to show a monotonic behavior over the whole investigated potential range, if the bias potential does apply completely across the whole space charge layer. According to the Gärtner-Butler model^36,37^ $I_{\mathrm{photo}}$ should depend on the electrochemical potential with $E^{1/2}$ after:

7 $$\begin{aligned} I_{\mathrm{photo}} = \hbox {e}P_{0}\alpha w = \hbox {e}P_{0} \sqrt{\frac{2\varepsilon \varepsilon _0(E-E_{\mathrm{fb}})}{qN_{\mathrm{d}}}}, \end{aligned}$$

with $P_{0}$ being the incident light power, $\alpha$ the light absorption coefficient, *w* the width of the space charge layer, $\varepsilon$ the relative dielectric constant of $\hbox {TiO}_2$, $\varepsilon _0$ the vacuum permittivity, *q* is the charge transferred per ion, $N_{\mathrm{d}}$ the doping density, and $E_{\mathrm{fb}}$ the flat band potential. However, the Gärtner-Butler model does not involve charge recombination in the depletion layer or pinning of the Fermi level by surface state charging. In the latter case the bias potential does not apply across the space charge layer, but across the Helmholtz layer, which is often corroborated by a plateau in the *E* versus $I_{\mathrm{photo}}$ curves. There is no obvious plateau observable in the LSV curves of the investigated $\hbox {TiO}_2$ samples, but especially the peculiar behavior of the 400 $^{\circ }\hbox {C}\ \hbox {TiO}_2$ nanolayer could be also understood in terms of a charging of surface states characterized by a rather broad energy distribution. E.g., at potentials more positive than the OCP the slope of the $j_{\mathrm{photo}}$ versus $(E-E_{\mathrm{fb}})^{1/2}$ curve changes strikingly at about $-0.6\,\hbox {V}$, $-0.5\,\hbox {V}$, and $-0.2\,\hbox {V}$. The 600 $^{\circ }\hbox {C}$ sample with its strongly sloped photocurrent might also be at least partially explained by Fermi level pinning caused by surface states with a rather broad distribution at lower energies. Interestingly, a similar nearly linear dependence of the photocurrent on potential was observed for thin film hematite photoelectrodes^38^. Klahr et al. explained this photocurrent slope by a very short hole drift length, which could be also an explanation for the behavior of the strained 600 $^{\circ }\hbox {C}\ \hbox {TiO}_2$ sample.

In order to support or refute the hypothesis of (bulk) defects in the different $\hbox {TiO}_2$ coatings we measured the wavelength dependent IPCE of all investigated $\hbox {TiO}_2$ samples. Photocurrents or high IPCE values at irradiation wavelengths in the visible range could indicate bulk defects, since in perfectly crystalline semiconductors only interband transitions can be excited at energies at least as high as the band gap energy. In contrast, the involvement of (defect) states within the band gap could give rise to electron transitions and hence photocurrents at lower irradiation energies. The results are shown in Fig. S3. In order to directly compare the spectra without being compromised by the very different absolute IPCE values, the values were normalized to the respective value at 365 nm of each sample. It can be clearly seen that the $\hbox {TiO}_2$ samples annealed at 300, 400 and 500 $^{\circ }\hbox {C}$ show the expected behavior of a semiconductor with a monotonically decreasing IPCE from UV to visible irradiation wavelengths. Though only the 500 $^{\circ }\hbox {C}$ sample was proven to be crystalline anatase, the other two samples might already have formed nanocrystalline structures with an at least partially continuous band structure. A closer look at the IPCE spectrum of the 300 $^{\circ }\hbox {C}$ $\hbox {TiO}_2$ might exhibit a slightly larger relative IPCE between 400 and 420 nm as compared to the 400 and $500\,^{\circ }\hbox {C}$ sample, which could point to a more strongly disturbed structure. In contrast, both the 200 and $600\,^{\circ }\hbox {C}$ sample exhibit significant photocurrents peaking at about 425 nm. The $\hbox {TiO}_2$ film annealed at $600\,^{\circ }\hbox {C}$ shows even features at higher wavelengths. These IPCE values can—as already mentioned—not be explained by a simple crystalline semiconductor. The $200\,^{\circ }\hbox {C}$ sample might be better characterized by a gel-like structure with the presence of OH groups and other molecular building blocks rather than by a crystalline semiconductor structure with delocalized bands. As for the $600\,^{\circ }\hbox {C}$ sample, high IPCE values in the visible range would support the interpretation, that the peculiar behavior of the $600\,^{\circ }\hbox {C}$ in the LSV curve with a rather low filling factor could be caused by charge trapping in band gap states. As already mentioned in the introductory chapter, deep donor or acceptor states in the band gap decrease the charge transfer rate since the electron hopping between deep traps is usually slower than migration through the conduction band. The finding also supports the observation of a strained, oxygen-deficient phase in the $600\,^{\circ }\hbox {C}$ sample as postulated to interpret the GIXRD results. In contrast, no absorption in the visible range was found for the $400\,^{\circ }\hbox {C}$ sample. Here, surface defects might be involved in the charge transfer processes as will be discussed below.

#### Chopped light chronoamperometry (CLCA)

**Figure 6:**
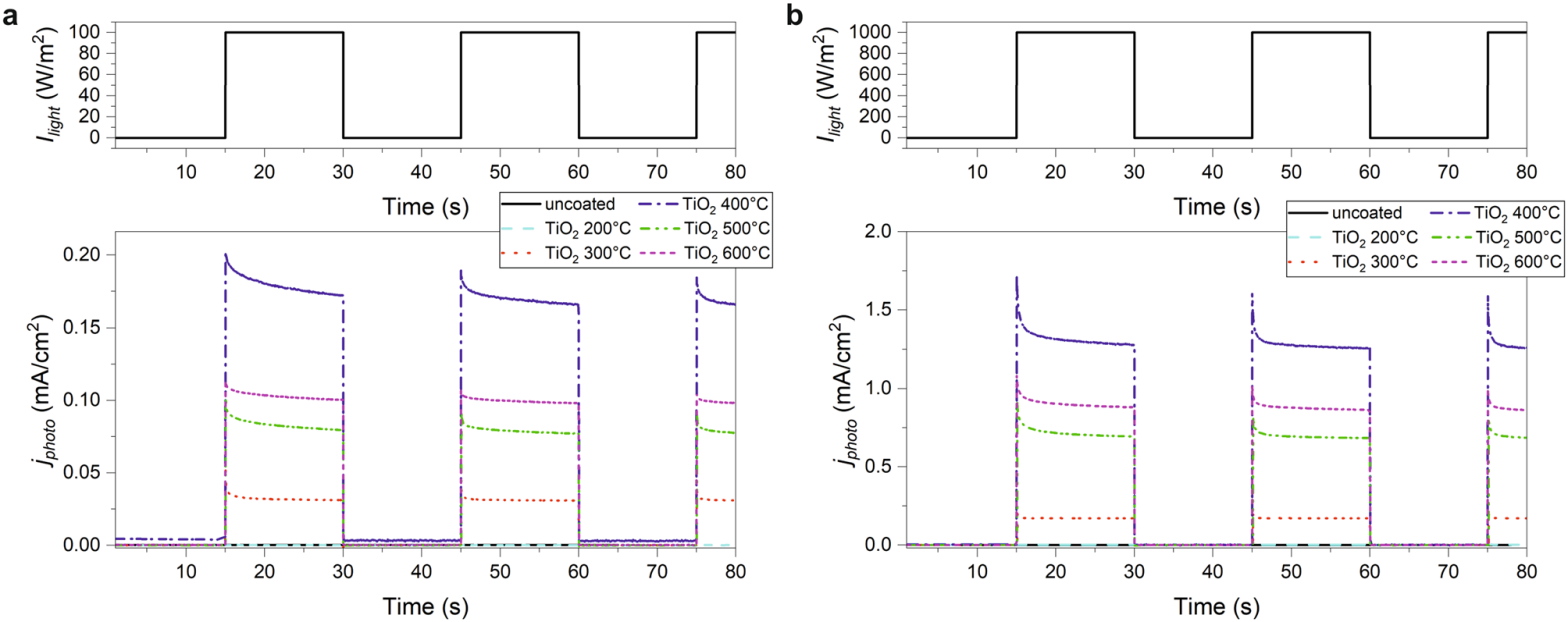
Chopped light chronoamperometric measurements at an applied potential of 0.1 V versus Ag/AgCl of neat and $\hbox {TiO}_2$-coated FTO glass slides calcined at temperatures indicated in the legend under (**a**) $100\,\hbox {W}/\hbox {m}^{2}$ and (**b**) $1000\,\hbox {W}/\hbox {m}^2$ irradiation at 369 nm.

The recombination at surface states can be investigated more closely by chronoamperometric measurements under chopped light conditions. The resulting photocurrent transients are reported in Fig. 6 under $100\,\hbox {W}/\hbox {m}^{2}$ (a) and $1000\,\hbox {W}/\hbox {m}^{2}$ (b) irradiation at 369 nm and at an applied potential of 0.1 V.

The absolute photocurrent intensities measured under irradiation reflect the results of the photopotentiodynamic measurements at higher potentials, i.e. the highest photocurrent is observed for the $400\,^{\circ }\hbox {C}\ \hbox {TiO}_2$ nanolayer, followed by the 600, 500, 300, $200\,^{\circ }\hbox {C}\ \hbox {TiO}_2$, and finally by the uncoated sample. Furthermore, the steady-state photocurrent measured just before the light was turned off was plotted versus the incident light intensity for all $\hbox {TiO}_2$ samples, resulting in a nearly ideal linear relationship (Fig. S4). These results are in agreement with the aforementioned Gärtner-Butler model for an illuminated semiconductor-liquid junction predicting a linear dependence between photocurrent and light intensity. However, the observed photocurrent transients depicted in Fig. 6 are all characterized by a spike after turning the light on followed by a slow decay of the photocurrent. This behavior has been assigned to a surface recombination mechanism^21^. After turning the light on, the photogenerated holes in titania diffuse to the surface of the layer, inducing a corresponding rather high photocurrent in the external circuit during the first seconds. Then, these holes get increasingly trapped by surface states. The thus initiated buildup of trapped holes acts as efficient recombination site for photogenerated electrons leading to a decrease of the initial photocurrent until a steady-state value is reached after a certain time. Corresponding time constants can be deduced from the photocurrent transients by applying the following two-phase exponential decay function:

8 $$\begin{aligned} j_{\mathrm{photo}} {=} j_0 + A_1\cdot \hbox {exp} [-(t-t_0)/\tau _1] + A_2\cdot \hbox {exp} [-(t-t_0)/\tau _2], \end{aligned}$$

where $j_0$ is the steady-state photocurrent density, $j_{\mathrm{photo}}$ is the photocurrent density at time *t*, $t_0$ is the time when the light is turned on, $A_1$ and $A_2$ are constants, and $\tau _1$ and $\tau _2$ are the time constants for the slow and the fast decay component, respectively. The obtained time constants for samples annealed at 300–$600\,^{\circ }\hbox {C}$ are summarized in Table S2. The photocurrent of the $200\,^{\circ }\hbox {C}$ sample was too small to obtain a reasonable fit. All other transients can be fitted by a faster component with time constants between 0.12 and 0.69 s, and a slower component with values between 2.37 and 9.04 s. An exception is the $300\,^{\circ }\hbox {C}\ \hbox {TiO}_2$ sample under $1000\,\hbox {W}/\hbox {m}^{2}$ irradiation with a time constant $\tau _2$ of 25.9 s. However, this component accounts for only 15.9% of the decay (ratio $A_2/(A_1 + A_2)$) and the decay of the latter sample could be also described with a reasonably good correlation by a one-phase exponential decay function. In contrast, the slow decay is the dominating component for the $400\,^{\circ }\hbox {C}$ (70.0%), $500\,^{\circ }\hbox {C}$ (52.7%), and $600\,^{\circ }\hbox {C}$ (64.6%) sample under $100\,\hbox {W}/\hbox {m}^{2}$ irradiation. Under the higher irradiation intensity of $1000\,\hbox {W}/\hbox {m}^{2}\ \tau _2$ decreases and its relative portion to the overall decay is diminished for the $400\,^{\circ }\hbox {C}$ (30.6%), $500\,^{\circ }\hbox {C}$ (41.2%), and $600\,^{\circ }\hbox {C}$ (38.8%) sample. Consequently, the surface recombination is accelerated with higher irradiation intensity. The highest decay time constants at $100\,\hbox {W}/\hbox {m}^{2}$ are observed for the $400\,^{\circ }\hbox {C}$ and $600\,^{\circ }\hbox {C}$ sample. The high influence of surface states on the photocurrent would be in agreement with the postulized Fermi level pinning by surface states discussed to explain the LSV measurements accompanied by the observation of rather low fill factors, which was especially apparent for the 400 and $600\,^{\circ }\hbox {C}\ \hbox {TiO}_2$ samples, too.

#### Intensity-modulated photocurrent (IMPS) and photovoltage spectroscopy (IMVS)

IMPS and IMVS are powerful techniques to extract photoelectrochemical kinetic constants for majority and minority charge carriers in semiconductors by applying a small sinusoidal perturbation to the incident light intensity^18,21,22,24,26,39^. In IMPS the photocurrent-time response is measured under closed-circuit conditions at a defined DC bias potential. At a bias potential more positive than the OCP the photogenerated majority carrier electrons diffuse to the current collecting back-contact of the $\hbox {TiO}_2$ film. The average electron transit time ($\tau _{\mathrm{D}}$) to the back-contact (or electron mobility) is given by the phase relationship between photocurrent perturbation and light modulation. It can be directly calculated from the complex Nyquist representation of the IMPS response:

9 $$\begin{aligned} \tau _{\mathrm{D}} {=} 1/\omega _{\mathrm{min,IMPS}}, \end{aligned}$$

with $\omega _{\mathrm{min},\mathrm{IMPS}}$ being the angular frequency of the minimum of the IMPS Nyquist semicircle. Moreover, absorbed photons generate a flux of minority carrier holes $J_{\mathrm{gen}}$ in the valence band toward the semiconductor-electrolyte interface. A part of the photogenerated holes diffuses with the flux $J_{\mathrm{ct}}$ to the semiconductor surface realizing an oxidation reaction via interfacial charge transfer. The quantum efficiency $\eta _{\mathrm{ct}}$ of the surface hole charge transfer process is defined as the quotient between hole charge transfer rate constant $k_{\mathrm{ct}}$ and the sum of $k_{\mathrm{ct}}$ and hole surface recombination constant $k_{\mathrm{rec}}$. $\eta _{\mathrm{ct}}$ can be deduced from the IMPS Nyquist plot by calculating the ratio between the low-frequency intercept (LFI) and high-frequency intercept (HFI) of the IMPS semicircle with the real axis:

10 $$\begin{aligned} \eta _{\mathrm{ct}} = \frac{J_{\mathrm{ct}}}{J_{\mathrm{gen}}} =\frac{k_{\mathrm{ct}}}{k_{\mathrm{ct}} + k_{\mathrm{rec}}} =\frac{\mathrm{LFI}}{\mathrm{HFI}}. \end{aligned}$$

$\eta _{\mathrm{ct}}$ is diminished by the competing flux $J_{\mathrm{rec}}$ of holes directly recombining with electrons from the conduction band at the semiconductor surface according to the following conversation equation:

11 $$\begin{aligned} J_{\mathrm{gen}} {=} J_{\mathrm{ct}} + J_{\mathrm{rec}}. \end{aligned}$$

The time constant $\tau _{\mathrm{t}}$ of this combined charge transfer and recombination process is the inverse of the sum of $k_{\mathrm{ct}}$ and $k_{\mathrm{rec}}$, and can be directly deduced from the angular frequency $\omega _{\mathrm{max},\mathrm{IMPS}}$ of the maximum of the IMPS Nyquist semicircle:

12 $$\begin{aligned} \omega _{\mathrm{max},\mathrm{IMPS}} {=} (\tau _{\mathrm{t}})^{-1} =k_{\mathrm{ct}} + k_{\mathrm{rec}}. \end{aligned}$$

Bulk majority carrier electron transport properties can be investigated with IMVS by measuring the photovoltage perturbation upon light modulation under open-circuit conditions. Here, the electron life time $\tau _{\mathrm{n}}$ is derived from the angular frequency of the minimum of the IMVS Nyquist semicircle $\omega _{\mathrm{min},\mathrm{IMVS}}$:

13 $$\begin{aligned} \tau _{\mathrm{n}} {=} 1/\omega _{\mathrm{min,IMVS}}. \end{aligned}$$

**Figure 7:**
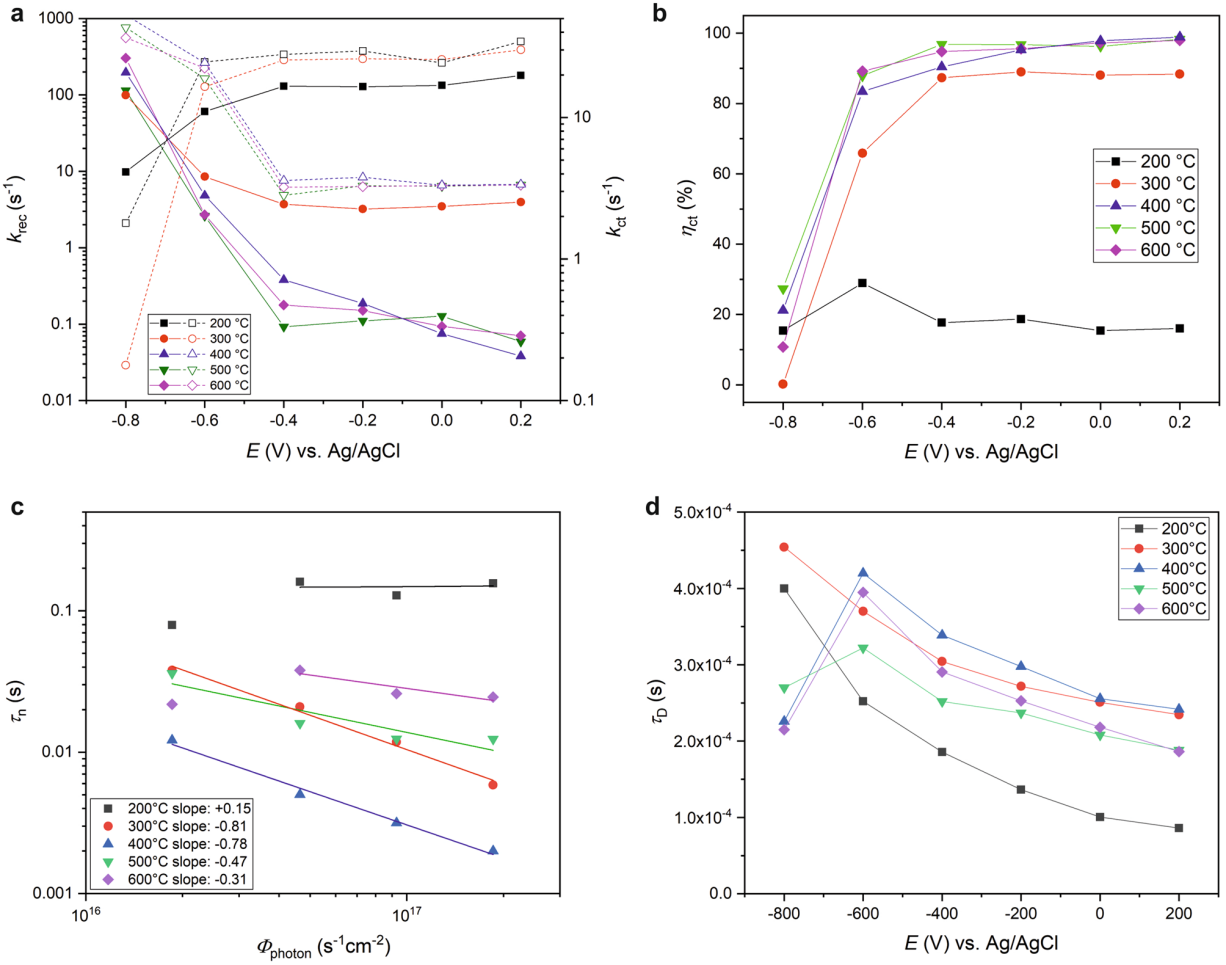
(**a**,**b**) Kinetic parameters as deduced from IMPS measurements with $k_{\mathrm{rec}}$ being the surface hole recombination rate (a, filled symbols), $k_{\mathrm{ct}}$ the surface hole charge transfer rate (**a**, open symbols), and $\eta _{\mathrm{ct}}$ the hole charge transfer efficiency (**b**) at different potentials. (**c**) Log-log plot of electron lifetime $\tau _{\mathrm{n}}$ versus incident photon flux $\Phi _{\mathrm{photon}}$ as retrieved from IMVS measurements. Solid lines represent linear least-square regressions of each data set with the obtained slopes given in the legend. (**d**) Electron transit times $\tau _{\mathrm{D}}$ for each $\hbox {TiO}_2$-coated FTO retrieved from IMPS measurements at different applied potentials and under 369 nm $100\,\hbox {W}/\hbox {m}^2$ irradiation. The drawn lines in (**a**,**b**,**d**) serve as guide to the eyes.

In panels a and b of Fig. S5 IMPS diagrams plotted in the complex plane (Nyquist representation) for the different $\hbox {TiO}_2$ films on FTO are exemplarily depicted for a DC bias potential close to the OCP ($-0.6\,\hbox {V}$) and for a positive voltage (+0.2 V), respectively. The high-frequency part of all circles is in the forth quadrant of the diagram (i.e. positive real and negative imaginary numbers) and $\omega _{\mathrm{min},\mathrm{IMPS}}$ at the minimum of the IMPS Nyquist semicircle can be easily deduced to calculate the average majority carrier electron transit time ($\tau _{\mathrm{D}}$) (Fig. 7d). In addition, all IMPS measurements are characterized by a low-frequency part lying in the first quadrant of the diagram (i.e. positive real and positive imaginary numbers). From this part the rate constants for the minority hole charge transfer and recombination can be deduced as described before. The respective rate constants are plotted in panel a of Fig. 7 for all applied bias potentials. All received recombination rates $k_{\mathrm{rec}}$ decrease with increasing potential except for the $200^{\circ }\hbox {C}$ sample, pointing for the other samples to a less dominant electron-hole recombination at potentials much more positive than the OCP. It becomes also obvious, that the recombination is with values below $0.1\,\hbox {s}^{-1}$ at 0.2 V versus Ag/AgCl slowest for the $400\,^{\circ }\hbox {C}$ sample, followed by the 500 and $600\,^{\circ }\hbox {C}\ \hbox {TiO}_2$. The ranking switches at potentials below 0 V, where the $500\,^{\circ }\hbox {C}\ \hbox {TiO}_2$ film now exhibits the smallest recombination rate, followed by the 600 and $400\,^{\circ }\hbox {C}$ sample, respectively. In contrast, the recombination is fastest for the $200\,^{\circ }\hbox {C}$, and second fastest for the $300\,^{\circ }\hbox {C}$ sample, reaching values that are nearly two or even three orders of magnitudes larger than the rates of the high temperature samples. A similar trend is observed for the charge transfer constant $k_{\mathrm{ct}}$. Here, the charge transfer is fastest for the samples annealed at the two lowest temperatures with values of 20–$40\,\hbox {s}^{-1}$ at potentials more positive than $-0.6\,\hbox {V}$. The 400, 500, and $600\,^{\circ }\hbox {C}$ samples can be hardly distinguished at potentials above $-0.6\,\hbox {V}$ exhibiting very similar values being approximately one order of magnitude smaller than the rates of the low temperature samples. With equation 10 the quantum efficiencies of the hole charge transfer process were calculated for each sample and the results are summarized in Fig. 7b. As could be already expected from the rate constants, the $\hbox {TiO}_2$ film annealed at $200\,^{\circ }\hbox {C}$ shows over the whole investigated potential range by far the lowest charge transfer efficiency with values of only about 20%. The $300\,^{\circ }\hbox {C}\ \hbox {TiO}_2$ shows better but still significantly worse quantum efficiencies than the rest of the samples, not achieving values exceeding 89%. The samples annealed at temperatures of 400, 500 and $600\,^{\circ }\hbox {C}$ exhibit the highest quantum efficiencies, reaching nearly 99% at 0.2 V. However, the $\hbox {TiO}_2$-coated FTO glass annealed at $400\,^{\circ }\hbox {C}$ catches up with the quantum efficiency of the other high temperature samples only at a potential of at least $-0.2\,\hbox {V}$, while it is significantly smaller at more negative potentials. This can be explained by the higher recombination rate and reflects also the potential dependent trends discussed for the LSV measurements.

Panels c and d of Fig. S5 show exemplarily the results of the IMVS measurements at light intensities of 100 and $1000\,\hbox {W}/\hbox {m}^{2}$, respectively. At the lower light intensity all samples give a clear semicircle in the complex Nyquist plane with nearly all values lying in the forth quadrant of the diagram. The angular frequency $\omega _{\mathrm{min},\mathrm{IMVS}}$ at the minimum can be easily identified to calculate the electron lifetime $\tau _{\mathrm{n}}$. However, the IMVS plots of the 500 and $600\,^{\circ }\hbox {C}\ \hbox {TiO}_2$ films at the higher light intensity are characterized by two not clearly separated semicircles, pointing to more than one charge recombination process. In these cases $\omega _{\mathrm{min},\mathrm{IMVS}}$ was still determined at the global minimum of the complex diagram to obtain the time constant for the (dominant) slower process at smaller frequencies. It is also remarkable that the IMVS diagrams of the 400, 500 and $600\,^{\circ }\hbox {C}$ samples show also values in the first quadrant of the complex plane. The determined electron lifetimes are plotted in Fig. 7c versus the incident photon flux. By applying a least-square linear regression for the approximately linear three or four data points of each sample, the slopes of the log-log electron lifetime-photon flux dependencies were deduced. Only the $200\,^{\circ }\hbox {C}$ shows a slightly positive slope, but due to the low data quality of the IMVS diagrams for this sample the interpretation is omitted. The 300 and $400\,^{\circ }\hbox {C}$ samples exhibit a slope of around $-0.8$. This value is close to $-1$, indicating that the reactions driven by electrons trapped in surface states are approximately first order in light intensity. In contrast, the slopes of the $500\,^{\circ }\hbox {C}$ and even more pronounced for the $600\,^{\circ }\hbox {C}$ sample are significantly smaller. Hence, these values indicate second order (theoretical slope: $-0.5$) or even higher order processes in light intensity. Finally, panel d of Fig. 7 shows the electron transit times in dependence of the DC bias potential as deduced from the IMPS diagrams. All electron transit times decrease with increasing potential at potentials above the OCP. Again, the values for the $\hbox {TiO}_2$ film annealed at $200\,^{\circ }\hbox {C}$ exhibit the largest deviations from the other samples with the lowest electron transit time at potentials more positive than the OCP. All other transit times are at least twice as high, at which the 300 and $400\,^{\circ }\hbox {C}$ samples generally show the highest electron transit times and thus smallest electron mobilities. Obviously, electron transit times are for all samples at least two orders of magnitude smaller than the electron lifetimes, indicating rather higher quantum efficiencies for the electron charge transfer process. In general, electron lifetimes and transit times are significantly smaller than the reciprocal hole charge transfer and recombination rates. This points to rate-determining hole charge transfer dynamics in all investigated $\hbox {TiO}_2$ samples. This would be in agreement with the results of Tang et al., who could show that the lifetime of photogenerated holes in $\hbox {TiO}_2$ is an important determinant for photocatalytic water splitting^19^.

#### Photoelectrochemical Impedance Spectroscopy (PEIS)

PEIS is another powerful method to study and understand the charge transfer in irradiated semiconductors in contact with an electrolyte. Klahr et al. suggested various physical models for the charge carrier dynamics in hematite photoelectrodes for water oxidation including the effects of surface states on the Fermi level and charge redistribution^40^. A typical equivalent circuit used to explain charge carrier dynamics in irradiated semiconductor-electrolyte junctions is depicted in Fig. 1c, with $R_{\mathrm{s}}$ being the $\hbox {TiO}_2/\hbox {FTO}$ (back-contact) resistance, $R_{\mathrm{ct},\mathrm{bulk}}$ the resistance for direct interfacial hole charge transfer via the valence band, $R_{\mathrm{trap}}$ the resistance for trapping of holes from the valence and electrons from the conduction band at surface states, $R_{\mathrm{ct},\mathrm{trap}}$ the resistance for charge transfer of holes via surface states, $C_{\mathrm{bulk}}$ is the capacitance associated with the space charge layer in series with the Helmholtz layer, and $C_{\mathrm{trap}}$ is the surface state capacitance. In this model $R_{\mathrm{ct},\mathrm{bulk}}$ can be associated with the rate constant $k_{1}$ and $R_{\mathrm{trap}}$ with the rate constants $k_{2}$ and $k_{4}$. In addition, the charge transfer via surface states resistance $R_{\mathrm{ct},\mathrm{trap}}$ can be associated with $k_{3}$, though a discrimination between $R_{\mathrm{ct},\mathrm{trap}}$ and $R_{\mathrm{ct},\mathrm{bulk}}$ just based on equivalent circuit fitting is often very challenging.

**Figure 8:**
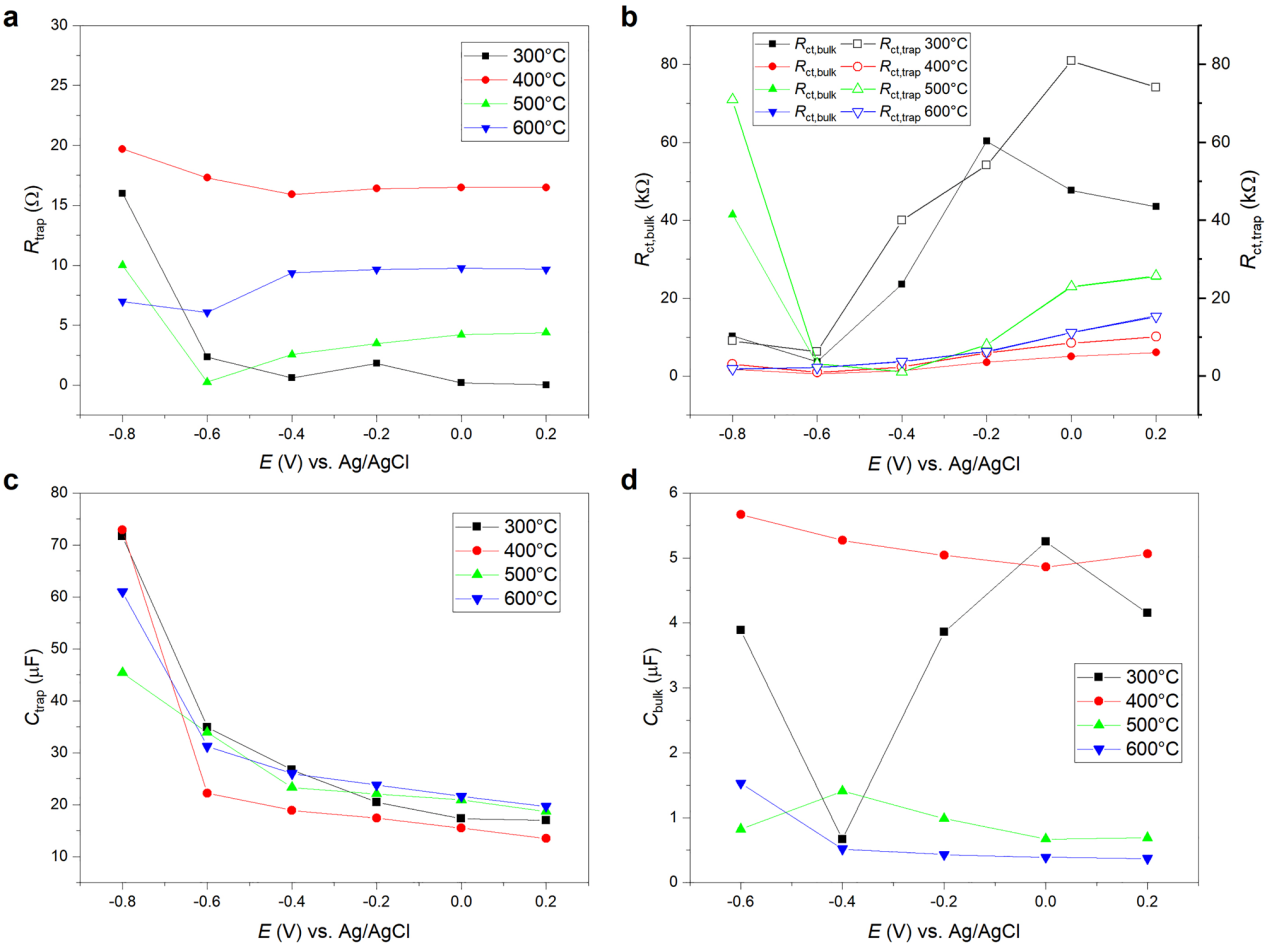
(**a**–**d**) Equivalent circuit element parameters extracted by fitting the PEIS data of $\hbox {TiO}_2$ nanolayers from Fig. S6 with the equivalent circuit shown in Fig. 1c in dependence on the applied potential. The drawn lines serve as guide to the eyes.

In Fig. S6 PEIS Nyquist plots for the $\hbox {TiO}_2$ nanolayers annealed at 300, 400, 500, and $600\,^{\circ }\hbox {C}$, respectively, and measured under $100\,\hbox {W}/\hbox {m}^2$ irradiation at potentials between − 800 and +200 mV are shown. PEIS results were analyzed by fitting the data with the equivalent circuit depicted in Fig. 1c. The $C_{\mathrm{bulk}}$ element was fitted by using a constant phase element (CPE) and the corresponding capacitances were calculated according to the equation derived by Brug et al.^41^. The thus extracted circuit element parameters were plotted versus the applied potentials and are presented in Fig. 8. The $200\,^{\circ }\hbox {C}$ sample is not further discussed since the obtained spectra were characterized by a low signal to noise ratio that could not be fitted very will with the discussed equivalent circuit. The $R_{\mathrm{s}}$ data are not shown since the obtained values were rather constant over the whole investigated potential range for all samples with magnitudes between about 7 and 13 $\Omega$, which is also consistent with an ohmic behavior of the $\hbox {FTO}/\hbox {TiO}_2$ interface.

In order to test the plausibility of the applied physical model the obtained resistances $R_{\mathrm{ct},\mathrm{bulk}}$ and $R_{\mathrm{ct},\mathrm{trap}}$ associated with direct charge transfer via the valence band and charge transfer via surface states, respectively, were compared with the resistance $R_{\mathrm{LSV}}$ obtained by the derivation of the linear sweep voltammetry curves from Fig. 5a according to:

14 $$\begin{aligned} R_{\mathrm{LSV}} = \left( \frac{\mathrm{d} I_{\mathrm{photo}}}{\mathrm{d}E}\right) ^{-1}. \end{aligned}$$

**Figure 9:**
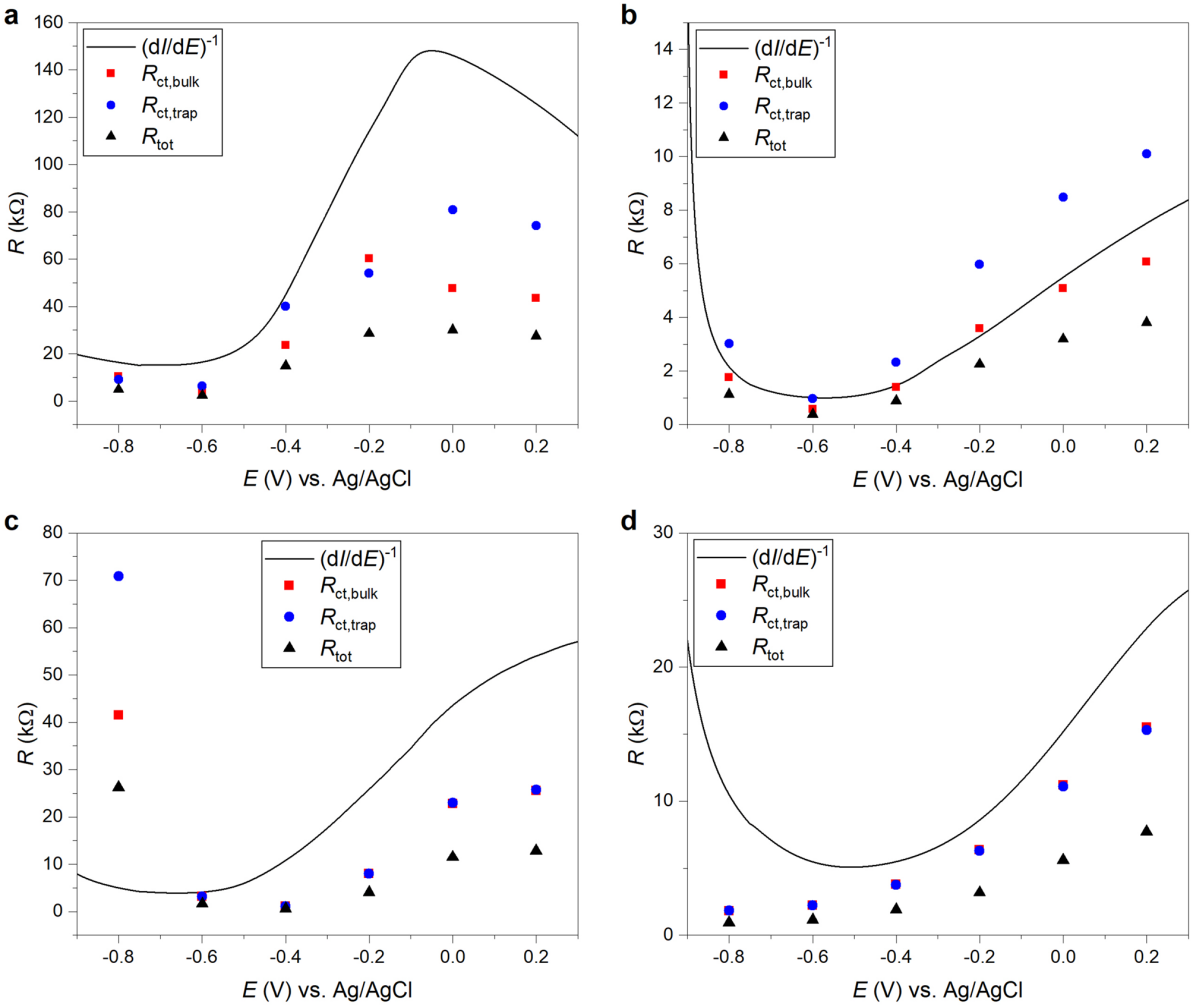
Comparison of resistances obtained by the derivation of linear sweep voltammetry curves after potential *E* with resistances deduced from Nyquist PEIS plots for $\hbox {TiO}_2$ nanolayers annealed at (**a**) $300\,^{\circ }\hbox {C}$, (**b**) $400\,^{\circ }\hbox {C}$, (**c**) $500\,^{\circ }\hbox {C}$, and (**d**) $600\,^{\circ }\hbox {C}$.

The summarized data in Fig. 9 show that all resistances follow similar trends with increasing potential for each sample. However, the resistances extracted from the PEIS data are systematically smaller than the resistances obtained by the derivation of the photocurrents. An exception is the $400\,^{\circ }\hbox {C}$ sample. Here, the $R_{\mathrm{ct},\mathrm{bulk}}$ values follow very well the derived photocurrents. We also calculated a total resistance $R_{\mathrm{tot}}$ by the addition of all serial resistances and parallel reciprocal resistances, respectively, as follows from the interconnection of the respective circuit elements in the applied equivalent circuit. Again, $R_{\mathrm{tot}}$ is systematically smaller than the derivative of the photocurrent. It should be also noted, that $R_{\mathrm{ct},\mathrm{trap}}$ and $R_{\mathrm{ct},\mathrm{bulk}}$ cannot be distinguished in the $500\,^{\circ }\hbox {C}$ and $600\,^{\circ }\hbox {C}$ sample, respectively. A fit of similar quality was also possible by completely omitting, e.g., the $R_{\mathrm{ct},\mathrm{trap}}$ element in the circuit resulting in a fit with a halved value for $R_{\mathrm{ct},\mathrm{bulk}}$ that also equals the $R_{\mathrm{tot}}$ value of the complete circuit (as expected for parallel resistances). Cachet and Sutter explained differences between the AC response and the DC photocurrent of $\hbox {TiO}_2$ by the contribution of very slow processes to the AC signal in the PEIS measurements at very low modulation frequencies^21^. This interpretation would be in agreement with the slow temporal decay of the photocurrents as observed in the CLCA measurements (cf. Fig. 6). If the physical model and former attribution of the resistances to the respective charge transfer processes is correct, the (faster) direct charge transfer of holes via the valence band would perfectly explain the photocurrent of the $400\,^{\circ }\hbox {C}$ sample. In contrast, the photocurrent of the 300 °C $\hbox {TiO}_2$ is in intensity and potential dependence closer to the surface state charge transfer associated with $R_{\mathrm{ct},\mathrm{trap}}$.

In addition, the magnitudes of the resistances deduced from the PEIS measurements can at least qualitatively explain the trends observed for the photocurrent curves of the different $\hbox {TiO}_2$ samples. The $300\,^{\circ }\hbox {C}$ sample exhibits the highest $R_{\mathrm{LSV}}$, $R_{\mathrm{ct},\mathrm{bulk}}$, $R_{\mathrm{ct},\mathrm{trap}}$ and $R_{\mathrm{tot}}$ values. Since these values are associated with the efficiency of the charge transport from the semiconductor to the solution, this in agreement with the lower $\eta _{\mathrm{ct}}$ value for this sample in comparison to the $\hbox {TiO}_2$ nanolayers annealed at higher temperatures and as extracted from the IMPS measurements (cf. Fig. 7b). Moreover, the measured absolute photocurrent increases at positive potentials versus Ag/AgCl in the direction $300\,^{\circ }\hbox {C}$, $500\,^{\circ }\hbox {C}$, $600\,^{\circ }\hbox {C}$ and $400\,^{\circ }\hbox {C}$, and this behavior is also well reflected by the deduced charge transfer resistances decreasing in the same direction. Since $R_{\mathrm{ct},\mathrm{bulk}}$ and $R_{\mathrm{ct},\mathrm{trap}}$ are associated with the rate constants $k_1$ and $k_3$, respectively, it is expected that these constants also increase in the direction $300\,^{\circ }\hbox {C}$, $500\,^{\circ }\hbox {C}$, $600\,^{\circ }\hbox {C}$, and $400\,^{\circ }\hbox {C}$.

As for the extracted capacitances, there is a significant difference between $C_{\mathrm{bulk}}$ of the $300\,^{\circ }\hbox {C}$ and $400\,^{\circ }\hbox {C}$ samples on the one hand and the 500 and $600\,^{\circ }\hbox {C}$ samples on the other hand. Since these quantities are associated with the capacitance of the space charge layer in series with the Helmholtz capacitance, this points to a very high charge accumulation and a slow diffusion and separation of charges in the space charge layer for the two samples annealed at lower temperatures. This would be in agreement with the relatively large electron transit time of these samples as deduced by the IMVS measurements (cf. Fig. 7d). Moreover, the $C_{\mathrm{trap}}$ values of the $400\,^{\circ }\hbox {C}$ sample above $-0.8\,\hbox {V}$ versus Ag/AgCl—and this also holds true at more positive potentials for the $300\,^{\circ }\hbox {C}$ sample—are systematically smaller than the respective values of the $500\,^{\circ }\hbox {C}$ and $600\,^{\circ }\hbox {C}$ samples. Since this capacitance is associated with the hole transfer ability to the electrolyte, this suggests that the $400\,^{\circ }\hbox {C}$ $\hbox {TiO}_2$ nanolayer (as well as the $300\,^{\circ }\hbox {C}$ sample at higher potentials) exhibits the lowest accumulation of holes at the semiconductor-electrolyte interface. This could be explained by effective surface hole transfer to the electrolyte in the case of the $400\,^{\circ }\hbox {C}$ sample and an efficient hole electron recombination at surface states in the case of the $300\,^{\circ }\hbox {C}$ sample. However, the $R_{\mathrm{trap}}$ resistances for the 300 and $400\,^{\circ }\hbox {C}$ samples do not agree very well. The quantities of the latter $\hbox {TiO}_2$ nanolayer are systematically higher over the whole investigated potential range, followed by the resistances of the $600\,^{\circ }\hbox {C}$, 500 and $300\,^{\circ }\hbox {C}$ samples. This parameter indicates a hampered trapping of holes from the valence and electrons from the conduction band at surface states and points to low corresponding rate constants $k_2$ and $k_4$ for the $400\,^{\circ }\hbox {C}$ sample, while this rate is highest for $\hbox {TiO}_2$ annealed at only $300\,^{\circ }\hbox {C}$. This interpretation would be in agreement with the observed order of the decay time constants in Table S2 deduced from the CLCA transients, which were also correlated with the respective hole recombination rate constants at surface states.

We also investigated the electrochemical impedance of all samples without irradiation to elucidate the influence of light on charge transfer. As a result, $R_{\mathrm{tot}}$ values extracted by fitting the experimental curves with the same equivalent circuit as the PEIS data are at least two orders of magnitude larger than the corresponding quantities under irradiation (e.g. $4.01\,\hbox {k}\Omega$ with $100\,\hbox {W}/\hbox {m}^2$ versus $249\,\hbox {k}\Omega$ without irradiation for the $500\,^{\circ }\hbox {C}$ sample at $-0.2\,\hbox {V}$ versus Ag/AgCl) pointing to a strongly hampered semiconductor-electrolyte charge transfer of the (intrinsic and extrinsic charge carriers) in the dark. This could be expected since only the irradation induced photovoltage provides a sufficient thermodynamic driving force for interfacial charge transfer. However, the $C_{\mathrm{trap}}$ values do not differ much in the presence or absence of light (e.g. for $500\,^{\circ }\hbox {C}$ sample at $-0.2\,\hbox {V}$: $22.1\,\upmu \hbox {F}$ with $100\,\hbox {W}/\hbox {m}^2$ versus $21.4\,\upmu \hbox {F}$ without irradiation) suggesting that irradiation does not significantly influence the charge accumulation at surface states. Moreover, the deduced $R_{\mathrm{trap}}$ values were slightly larger in the absence of light (e.g. for $500\,^{\circ }\hbox {C}$ sample at $-0.2\,\hbox {V}$: $3.47\,\Omega$ with $100\,\hbox {W}/\hbox {m}^2$ versus $4.26\,\Omega$ without irradiation), but the $C_{\mathrm{bulk}}$ parameters were very similar (e.g. for $500\,^{\circ }\hbox {C}$ sample at $-0.2\,\hbox {V}$: 983 nF with $100\,\hbox {W}/\hbox {m}^2$ versus 999 nF without irradiation). This points to an accelerated trapping of charge carriers in surface states, but to nearly no increase of charge carriers in the space charge layer upon irradiation.

## Discussion

Since the structural analysis has shown, that only the $\hbox {TiO}_2$ samples annealed at temperatures of at least $500\,^{\circ }\hbox {C}$ are perfectly crystalline, it becomes obvious that the presence of crystalline anatase is not necessarily a prerequisite for both a high photocatalytic and photoelectrocatalytic activity. Instead, the $400\,^{\circ }\hbox {C}$ sample showing no distinct reflections in the diffractogram exhibits similarly high photocurrents and MB degradation rates as the crystalline anatase samples annealed at 500 and $600\,^{\circ }\hbox {C}$, respectively.

However, electron-hole recombination constants as deduced by IMPS can serve as discriminator between samples with high and low photoactivities. Hence the 200 and $300\,^{\circ }\hbox {C}$ $\hbox {TiO}_2$ films are characterized by an exceedingly fast electron-hole recombination. In addition, the resulting hole charge transfer efficiencies are significantly lower than in other samples, since the very effective recombination processes cannot be balanced by the rather high charge transfer rates. The low IPCE, photocurrents, MB degradation rates and antibacterial activity of the low temperature samples can hence be explained by the dominating recombination of photogenerated holes with electrons. This interpretation is further supported by the highest semiconductor-electrolyte charge transfer resistance of all samples as extracted from the AC response upon irradiation.

The samples annealed at 400, 500, and $600\,^{\circ }\hbox {C}$ show very similar MB degradation rates, but a very different photopotentiodynamic behavior. The former sample is characterized by the highest IPCE at very positive potentials. However, at potentials close to the OCP the photocurrent and IPCE are similar or even smaller than observed for the $500\,^{\circ }\hbox {C}$ sample. This potential dependent effect can be explained by the respective potential dependent hole charge transfer dynamics of the different samples. The surface hole recombination rate $k_{\mathrm{rec}}$ is higher and the resulting hole charge transfer efficiency $\eta _{\mathrm{ct}}$ is smaller for the $400\,^{\circ }\hbox {C}$ than for the 500 and $600\,^{\circ }\hbox {C}$ samples at potentials closer to the OCP. Only at potentials above $-0.2\,\hbox {V}$ the $400\,^{\circ }\hbox {C}$ sample can catch up with the respective values of the 500 and $600\,^{\circ }\hbox {C}\ \hbox {TiO}_2$ layers or does even outperform them. In this potential range the photocurrent and IPCE order of the samples switches as well. Hence the lower photoactivity of the $400\,^{\circ }\hbox {C}\ \hbox {TiO}_2$ at more negative potentials can be explained by a rather strong charge recombination of surface holes. This interpretation is supported by the rather small fill factor, low electron lifetime $\tau _{\mathrm{n}}$ and exceptionally high electron transit time $\tau _{\mathrm{D}}$ at negative potentials. This would be in agreement with the absence of crystalline features in the diffractogram pointing to a still high density of (surface) defect states in the $400\,^{\circ }\hbox {C}$ nanolayer. Small semi- or nanocrystalline domains might have been already formed at this intermediate temperature (though not observable in the diffractogram due to extreme peak broadening) giving rise to the much higher photoactivity as compared to the 200 and $300\,^{\circ }\hbox {C}$ sample. Moreover, the very dominant but slow photocurrent decay in the CLCA transient experiments—which was interpreted in terms of a recombination of photogenerated holes with surface states—further supports the presence of a highly reactive surface. In addition, the OCP is slightly more positive than the respective values of the 500 and $600\,^{\circ }\hbox {C}$ samples, pointing to a lower thermodynamic driving force to partipate in redox reactions. In contrast, the highest observed photocurrent at very positive potentials would be in agreement with the lowest charge transfer resistance of all samples. Hence a very high charge transfer rate $k_1$ for direct hole transfer via the valence band to the electrolyte can be expected for this sample. The $600\,^{\circ }\hbox {C}$ sample is characterized by a slightly reduced MB degradation rate, a shift of the OCP to more positive values and a lower photocurrent at negative potentials versus Ag/AgCl as compared to the respective values of the $500\,^{\circ }\hbox {C}$ sample. At negative potentials $k_{\mathrm{rec}}$ is slightly higher than the respective rate constant of the sample annealed at $500\,^{\circ }\hbox {C}$. This observation could give rise to a similar explanation in terms of an increased surface hole recombination rate as already discussed for the $400\,^{\circ }\hbox {C}\ \hbox {TiO}_2$. Moreover, the wavelength-dependent IPCE measurement provided hints for bulk defects that might also hamper an efficient charge transfer. The charge transfer resistance at positive potentials is smaller than the value of the $500\,^{\circ }\hbox {C}\ \hbox {TiO}_2$, but still larger than the respective resistance of the $400\,^{\circ }\hbox {C}$ sample, giving a straightforward explanation of the photocurrent ranking observed for these samples at positive potentials. A further point of discrimination is the rather high trapping resistance $R_{\mathrm{trap}}$ of the $600\,^{\circ }\hbox {C}$ sample, which shows the second highest value at potentials more positive than $-0.6\,\hbox {V}$, succeeding the respective resistance of the $400\,^{\circ }\hbox {C}$ sample. Since this resistance is interpreted in terms of the efficiency of the trapping of holes from the valence band and electrons from the conduction band in surface states, it points to rather small rate constants $k_2$ and $k_4$, respectively, at positive potentials, being in agreement with the slow photocurrent transients of the CLCA measurements (where the magnitude of the decay time constants of the $500\,^{\circ }\hbox {C}$ sample also succeeds the $400\,^{\circ }\hbox {C}$ sample at 0.1 V). Obviously, this sample exhibits properties that compare better with the $400\,^{\circ }\hbox {C}$ than with the $500\,^{\circ }\hbox {C}$ sample. The XRD pattern points to a strained elementary cell of anatase upon annealing at $600\,^{\circ }\hbox {C}$, probably in combination with the formation of oxygen-deficient phases. This process is very likely accompanied by the formation of new defects and surface states that can explain the peculiar photo(electro)catalytic properties of this sample.

In conclusion, it could be shown by a combined photocatalytic, antimicrobial, and photoelectrochemical study that the respective photoactivities of amorphous and crystalline $\hbox {TiO}_2$ nanolayers can be best correlated, if the extracted photoelectrochemical parameters such as charge transfer and recombination rates, charge transfer efficiencies and resistances, photocurrents as well as IPCE values are measured at potentials close to the OCP, which has been summarized in Fig. 5b by directly comparing the photocatalytic and photoelectrocatalytic trends in one graph. Hence interfacial charge transport parameters at the OCP as determined by photoelectrochemical measurements can be used indeed as descriptors for predicting and understanding the photocatalytic activity of $\hbox {TiO}_2$ coatings.

## Methods

### Synthesis of $\hbox {TiO}_2$ thin films

Titania thin films were prepared by a sol–gel spin-coating method. First, 150.0 mL isopropanol (for analysis, Sigma Aldrich) was cooled in a beaker to about $0\,^{\circ }\hbox {C}$ in a a sodium chloride/ice bath. 1.8 mL tetraisopropyl orthotitanate (for synthesis, Sigma Aldrich) was slowly added with a dropping funnel to the stirred solution. Subsequently, 0.5 mL nitric acid ($65\,\%$, for analysis, Sigma Aldrich) was added dropwise with a pipette. The resulting sol was aged for 60 minutes at about $0\,^{\circ }\hbox {C}$. The titania thin films were deposited on glass slides with a fluorine-doped tin oxide (FTO) coating (surface resistivity $7\,\Omega /\hbox {sq}$, Sigma Aldrich). 25 mm × 25 mm FTO glass slides were first successively cleaned by ultrasonic treatments in acetone, ethanol and double deionized water (Milli-Q Integral 3 system, $18.3\,\hbox {M}\Omega$) for about 5 min each. After drying under ambient conditions, a cleaned FTO glass slide was transferred to a spin coater with vacuum fixation (SCE 150, Quantum Design). Three drops of the titania sol were added onto the FTO side (or on the opposite, non FTO-side for GIXRD measurments) of the glass slide while applying a rotation speed of 60 rps. After 60 seconds the spin coating was stopped and the glass slide was successively dried under ambient conditions for 1 hour. The spin-coating procedure was repeated for two times for each glass slide. Afterwards, the coated glass was cleaned by ultrasonic treatments in aceton, ethanol and double-deionized water, respectively, for 2 minutes in each solvent. Subsequently, the coated glass slides were annealed in ambient air for two hours in a muffle furnace (L5/11/B410, Nabertherm) at 200, 300, 400, 500, and $600\,^{\circ }\hbox {C}$, respectively.

### Structural characterization

$\hbox {TiO}_2$ nanolayers coated on the FTO-coated side and on the opposite non-FTO-coated side of quartz glass samples, respectively, were analyzed with grazing incidence X-ray diffraction (GIXRD) by using the Malvern Panalytical XPertPro diffractometer with a grazing incidence setup. $\hbox {Cu}\ \hbox {K}_\alpha$ radiation was applied at an operation voltage of 45 kV and a current of 40 mA. Data were collected with an incident angle of $0.5^{\circ}$ and with a scan rate of $0.01^{\circ}/\hbox {s}$ in the $2\theta$ range between 10 and $70^{\circ}$. Field emission scanning electron microscopy (FESEM) and energy-dispersive X-ray spectroscopy (EDX) were performed by preparing cross-sections of the quartz $\hbox {glass}/\hbox {FTO}/\hbox {TiO}_2$ samples and by using a Hitachi S4800 microscope.

### Photodegradation of methylene blue

To determine the degradation rate an aqueous solution of methylene blue (Sigma Aldrich) with a concentration of $10\,\upmu \hbox {mol}/\hbox {L}$ was prepared. To enable a direct comparison with the photoelectrochemical experiments the same light source Zahner LED LS365-2 and photoelectrochemical cell PECC-2 (Zahner) was used for the experiments. 7 mL of the methylene blue solution was inserted into the photoelectrochemical cell PECC-2, which also contained the uncoated and $\hbox {TiO}_2$-coated FTO glass slides, respectively. The irradiation experiments were executed inside a light-exclusion box. After a waiting and equilibration time of 30 minutes—in order to minimize adsorption effects—a first 2.5 mL sample of the methylene blue solution in the PECC-2 was taken with a syringe and inserted into a 1 cm quartz cuvette. The absorbance of the sample at 664 nm was measured immediately in the UV-Vis spectrometer Specord 200 (Analytik Jena). This value served as reference for all the following measurement points after irradiation to obtain the relative concentration $\hbox {c}/\hbox {c}_0$ in dependence on irradiation time. Subsequently, the methylene blue sample was transferred back into the PECC-2, and the irradiation of the glass slide in the PECC-II was started by using the calibrated LED LS365-2 (Zahner) at a potentiostat/silicon diode sensor controlled light intensity of $1000\,\hbox {W}/\hbox {m}^{2}$. 2.5 mL samples of the methylene blue solution in the PECC-2 were taken every two minutes irradiation time to measure the absorbance in dependence of irradiation time as described before. During sampling and measuring absorption spectra, the light source was turned off to stop further light-induced degradation in the photoelectrochemical cell. After the absorption measurement, the sample was subsequently transferred back into the PECC-2 again, the LED was turned on again and this procedure was repeated until a total irradiation time of 20 mins was reached. All degradation curves for each sample were measured three times with fresh methylene blue solution to calculate the mean and the standard error.

### Photocatalytic antibacterial activity tests

Escherichia coli XL1-Blue strain was used as model organism to assess the antimicrobial properties of $\hbox {TiO}_2$-coated FTO glass slides. For this study test series with uncoated and $\hbox {TiO}_2$-coated FTO glass slides calcined at 300 and $500\,^{\circ }\hbox {C}$, respectively, were investigated. Overnight cultures of *E. coli* bacteria were grown in 5 mL Luria Bertani (LB) agar at $38\,^{\circ }\hbox {C}$. The investigated FTO glass slides were sterilized for 1.5 h at $180\,^{\circ }\hbox {C}$. Subsequently, $50\,\upmu \hbox {L}$ of the E. coli overnight culture (100 times diluted with LB medium) was added onto the glass slides and evenly distributed with an inoculating loop. The bacteria-coated glass slides were placed in the Vilber CN-6 dark room and irradiated with the UV-A lamp VL-6.L (6 W power, Vilber) exhibiting a peak wavelength of 365 nm. Non-irradiated glass slides of each test series analyzed after 120 min in the dark served as control. For each test series glass slides were removed from the dark/UV chamber after 30, 60, 90, and 120 min, respectively. The surviving bacteria on the glasses after each irradiation time series were immediately collected by purging the glass slides with $450\,\mu \hbox {L}$ of an aqueous sodium chloride solution ($0{,}9\,\%$) into a Petri dish. The procedure was repeated four times using always the same purge solution. The received purge solution was diluted with 4.05 mL aqueous sodium chloride solution ($0{,}9\,\%$) and cultured on agar plates for 24 hours at $38\,^{\circ }\hbox {C}$. The number of surviving bacteria was counted and is expressed as the logarithm of the ratio (survival ratio) of the number of viable bacteria remaining after exposure to experimental conditions (S) to the number of the initial viable bacteria ($\hbox {S}_0$) ($\hbox {log}\ \hbox {S}/\hbox {S}_0$).

### Photoelectrochemical characterization

Photoelectrochemical measurements were utilized with a Zahner Zennium electrochemical workstation in connection with a Zahner PP211 potentiostat for light control. The investigated FTO glass slide was contacted as working electrode with a stainless steel mesh in a Kel-F (PCTFE)-based gas-tight photoelectrochemical cell PECC-2 (Zahner) in transparent working electrode configuration with two quartz windows. For the standard three electrode configuration a platinum wire was used as counter electrode and all potentials were measured against and referred to a Ag/AgCl reference electrode. Due to the geometry of the cell a constant circular area with a diameter of 1.8 cm of the glass sample was always irradiated. All electrochemical measurements were carried out in aqueous 1.0 M NaOH electrolyte. After installing the glass slide into the photoelectrochemical cell, but before the electrochemical measurements, the electrolyte was deaerated with nitrogen gas. The photoelectrochemical studies were performend in the dark and with a calibrated UV light LED (LS365-2; dominant wavelength $\lambda =369\,\hbox {nm}$) providing DC light intensities up to $2000\,\hbox {W}/\hbox {m}^{2}$, respectively. The Zahner TLS03 tunable UV-Vis light source was used for wavelength dependent IPCE measurements. The incident light intensity on the sample was controlled with a calibrated silicon diode photosensor. The sensor output was fed into the Zahner Zennium/Potentiostat PP211 control loop for controlling the light intensity and modulation of the LED. All photoelectrochemical experiments were performed inside a light-exclusion box/Faraday cage.

## Supplementary Information

Supplementary Information.
