# Supplementary material for: Investigating and correlating photoelectrochemical, photocatalytic, and antimicrobial properties of TiO2 nanolayers
Source: Sci Rep. 2021 Nov 12;11:22200. doi: 10.1038/s41598-021-01165-x (PMC8589999; doi:10.1038/s41598-021-01165-x)
Supplement: Supplementary file 1 — Supplementary Information. [file 41598_2021_1165_MOESM1_ESM.pdf]

Supplementary Information:

Investigating and correlating photoelectrochemical, photocatalytic, and antimicrobial properties of TiO_2 nanolayers

Volker Seiß[1], **Uta Helbig**[2], **Ralf Lösel**[1], **and Maik Eichelbaum**[1,*]

[1]Georg Simon Ohm University of Applied Sciences Nuremberg, Faculty of Applied Chemistry, Nuremberg, 90489, Germany

[2]Georg Simon Ohm University of Applied Sciences Nuremberg, Faculty of Materials Engineering, Nuremberg, 90489, Germany

[*]maik.eichelbaum@th-nuernberg.de

Supplementary Tables and Figures

	uncoated	TiO_2 200°C	TiO_2 300°C	TiO_2 400°C	TiO_2 500°C	TiO_2 600°C
R_{MB} (ppm/min)	0.0074	0.022	0.030	0.046	0.047	0.042

Table S1. Methylene blue degradation rates R_{MB} for uncoated and TiO_2-coated FTO quartz glass samples annealed at the indicated temperatures.

Time constants	TiO_2 300°C	TiO_2 400°C	TiO_2 500°C	TiO_2 600°C
τ_1/s (100 W/m^2)	0.12	0.69	0.26	0.41
τ_2/s (100 W/m^2)	2.37	9.04	6.54	8.40
τ_1/s (1000 W/m^2)	0.40	0.30	0.33	0.34
τ_2/s (1000 W/m^2)	25.9	4.26	4.04	4.88

Table S2. Extracted time constants for photocurrent transients of TiO_2-coated FTO glasses measured by chopped light chronoamperometry at 0.1 V versus Ag/AgCl and under irradiation at 369 nm.

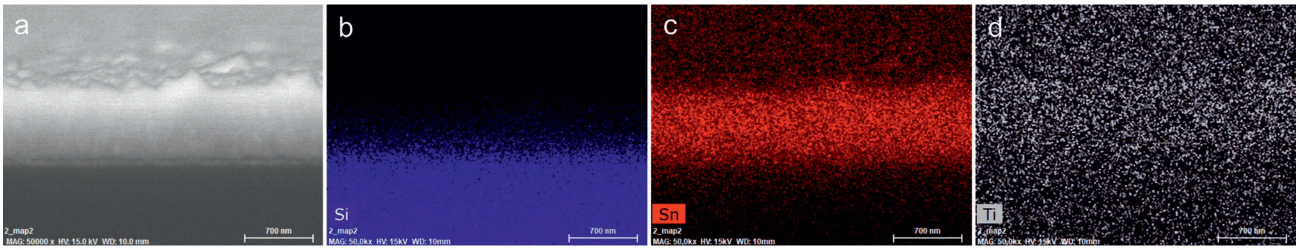

Figure S1. Cross-sectional field-emission scanning electron microscopy (FESEM) image of a TiO_2 coated FTO glass calcined at 200°C (a) and corresponding EDX elemental maps for silicon (b), tin (c) and titanium (d).

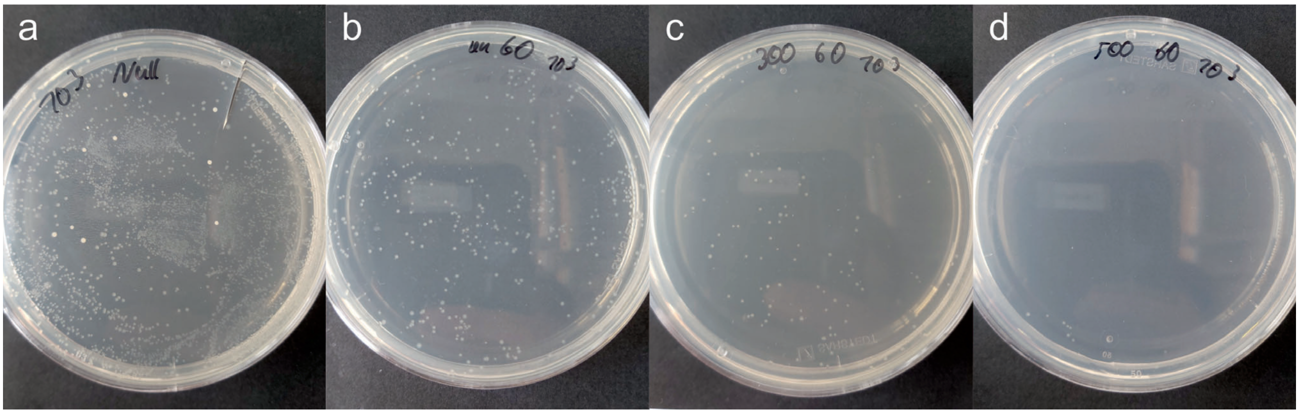

Figure S2. Images of petri dishes with bacterial cultures treated under different experimental conditions after culturing and plate pouring: a) Blank sample without further treatment. b) Sample collected after 60 minutes UV-A irradiation on neat FTO glass. c) Sample collected after 60 minutes UV-A irradiation on TiO_2-coated FTO glass calcined at 300°C. d) Sample collected after 60 minutes UV-A irradiation on TiO_2-coated FTO glass calcined at 500°C.

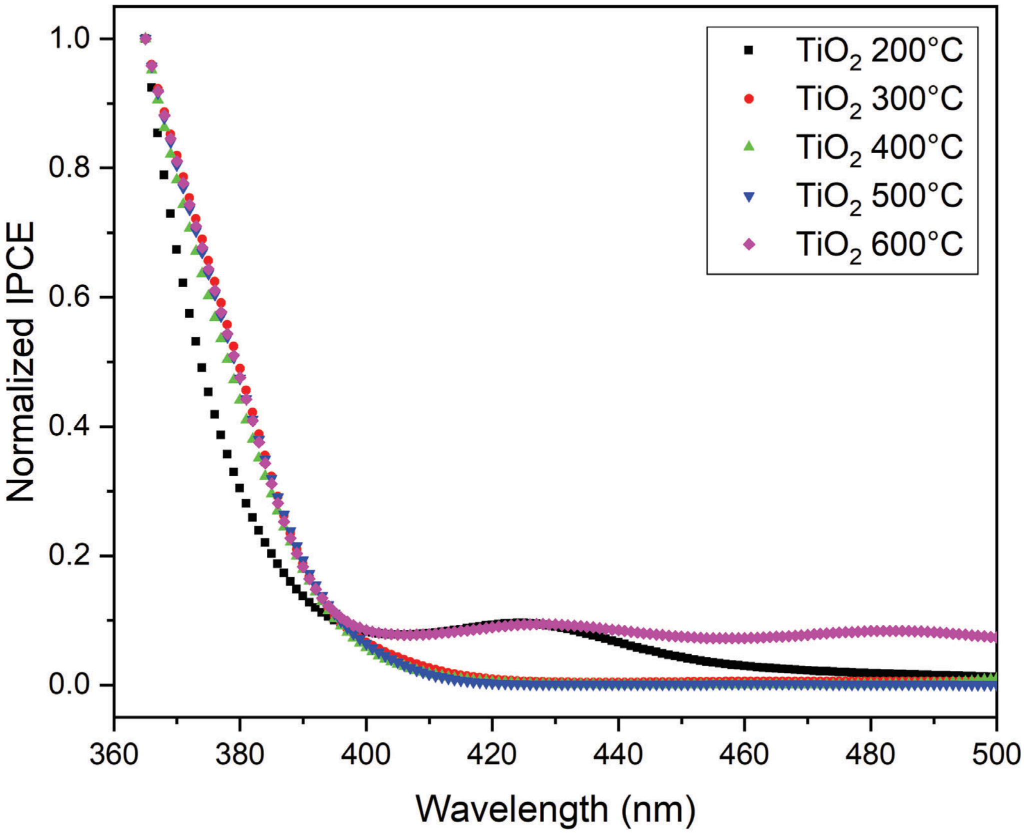

Figure S3. Wavelength dependent IPCE of TiO_2-coated FTO normalized to the maximum IPCE value at 365 nm measured at light intensities of 100 W/m^2 and a potential of 500 mV versus Ag/AgCl reference electrode.

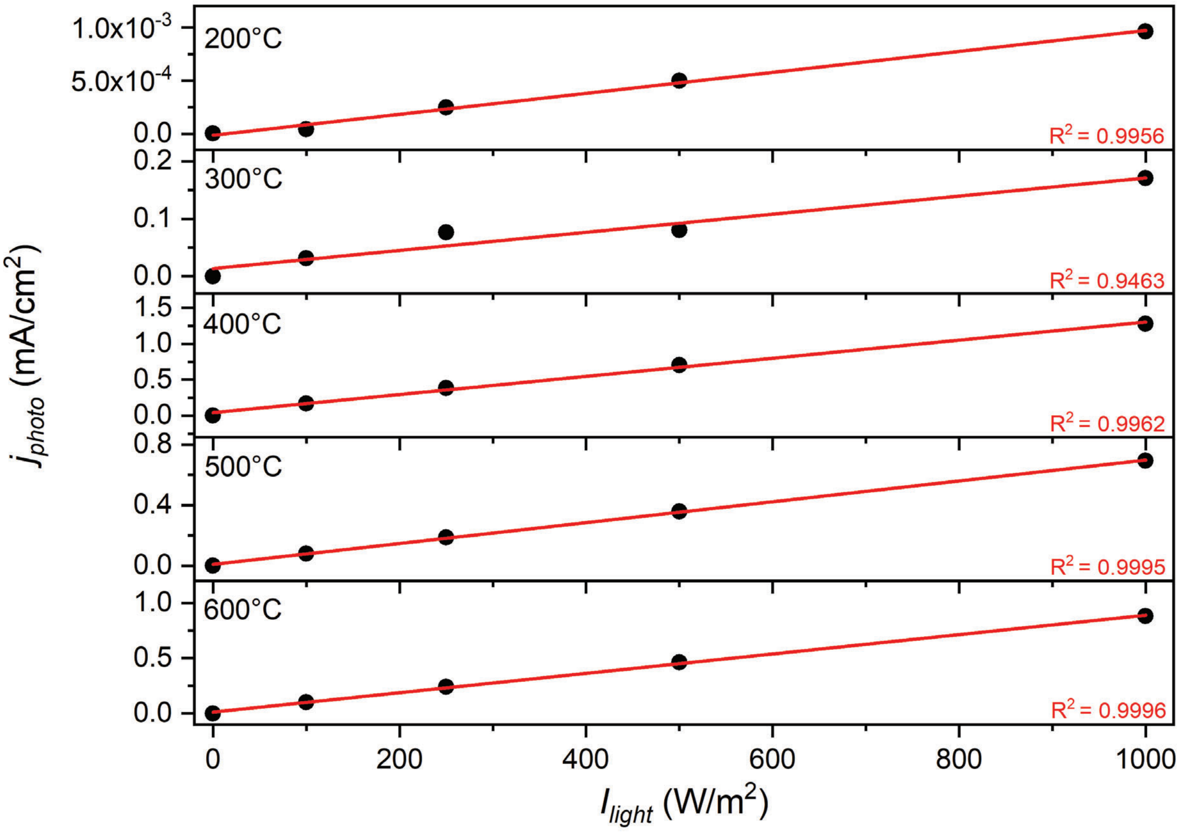

Figure S4. Plot of steady-state photocurrent densities versus light intensity for TiO_2-coated FTO glass slides calcined at temperatures indicated in the legend under irradiation at 369 nm and at a potential of 0.1 V versus Ag/AgCl. The red lines are the results of linear least-square regressions with R^2 being the resulting coefficients of determination.

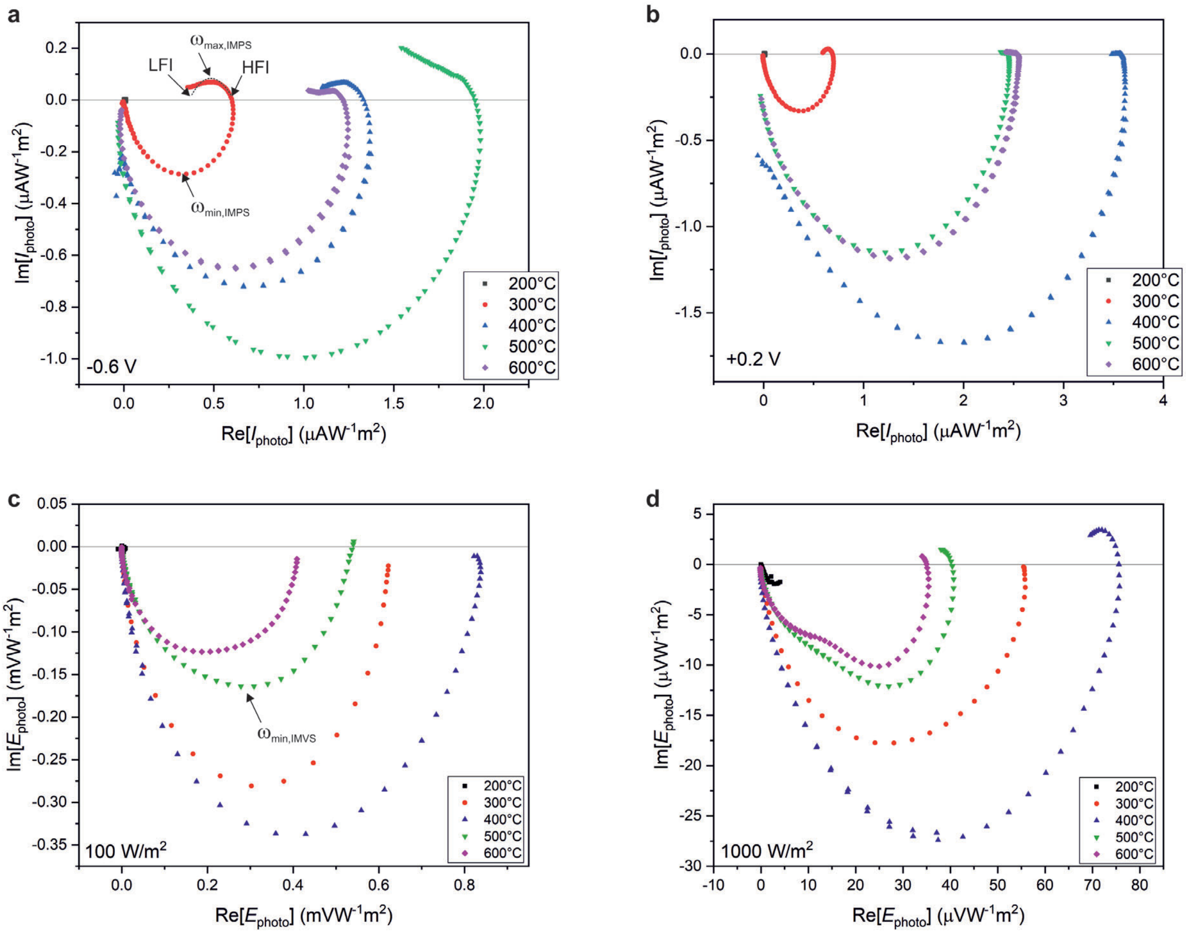

Figure S5. a-b) Representative IMPS Nyquist plots of TiO_2 coated FTO annealed at different temperatures under modulated 369 nm irradiation measured at -0.6 V (a) and +0.2 V versus Ag/AgCl (b). For the 300°C IMPS curve in a) the principal kinetic analysis is indicated by determining the high frequency intercept (HFI) and low frequency intercept (LFI) with the real axis, the angular frequencies at maximum $\omega_{\mathrm{max,IMPS}}$ and minimum $\omega_{\mathrm{min,IMPS}}$ of the plot, where the dashed black line is an extrapolation of the curve to estimate the LFI. c-d) Representative IMVS Nyquist plots of TiO_2 coated FTO annealed at different temperatures under modulated 369 nm irradiation measured at 100 W/m^2 (c) and 1000 W/m^2 (d). For the 500°C IMVS curve in c) the principal kinetic analysis is indicated by determining the angular frequency at the minimum ($\omega_{\mathrm{min,IMVS}}$) of the plot.

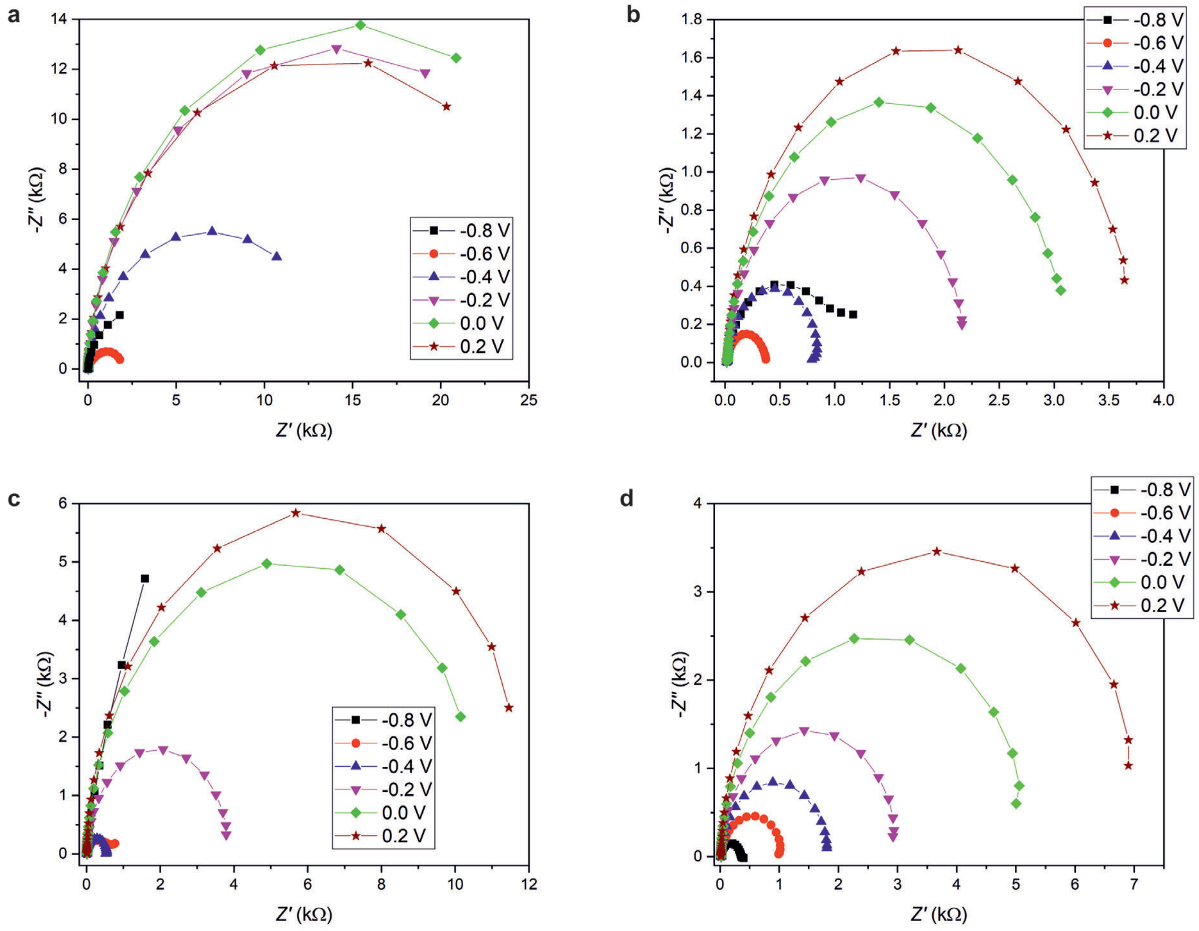

Figure S6. PEIS Nyquist plots of TiO_2-coated FTO annealed at a) 300°C, b) 400°C, c) 500°C, and d) 600°C under 369 nm irradiation at 100 W/m^2 measured at different applied potentials versus Ag/AgCl. The drawn lines serve as guide to the eyes.
